# Novel liquid biopsy CNV biomarkers in malignant melanoma

**DOI:** 10.1038/s41598-024-65928-y

**Published:** 2024-07-09

**Authors:** E. Lukacova, Z. Hanzlikova, P. Podlesnyi, T. Sedlackova, T. Szemes, M. Grendar, M. Samec, T. Hurtova, B. Malicherova, K. Leskova, J. Budis, T. Burjanivova

**Affiliations:** 1 Department of Molecular Biology and Genomics, Comenius University in Bratislava, Jessenius Faculty of Medicine in Martin (JFM CU), Martin, Slovakia; 2 Geneton Ltd., Bratislava, Slovakia; 3 Instituto de Investigaciones Biomedicas de Barcelona (IIBB), CSIC /Centro Investigacion Biomedica en Red Enfermedades Neurodegenerativas (CiberNed), Barcelona, Spain; 4 Science Park, Comenius University in Bratislava, Bratislava, Slovakia; 5 Laboratory of Bioinformatics and Biostatistics, Biomedical Center Martin, Comenius University in Bratislava, Jessenius Faculty of Medicine in Martin (JFM CU), Martin, Slovakia; 6 Department of Medical Biology, Jessenius Faculty of Medicine in Martin, Comenius University in Bratislava, Martin, Slovakia; 7 Department of Dermatovenereology, Jessenius Faculty of Medicine in Martin, Comenius University in Bratislava, Martin, Slovakia; 8 Department of Clinical Biochemistry, University Hospital in Martin and Jessenius Faculty of Medicine, Comenius University, Martin, Slovakia; 9 Department of Pathological Anatomy, Jessenius Faculty of Medicine and University Hospital in Martin, Comenius University, Martin, Slovakia

**Keywords:** Melanoma, Liquid biopsy, ctDNA, Biomarker, ddPCR, lcWGS, Cancer, Computational biology and bioinformatics, Molecular biology, Biomarkers, Molecular medicine, Oncology

## Abstract

Malignant melanoma (MM) is known for its abundance of genetic alterations and a tendency for rapid metastasizing. Identification of novel plasma biomarkers may enhance non-invasive diagnostics and disease monitoring. Initially, we examined copy number variations (CNV) in *CDK* genes (*CDKN2A*, *CDKN2B*, *CDK4*) using MLPA (gDNA) and ddPCR (ctDNA) analysis. Subsequently, low-coverage whole genome sequencing (lcWGS) was used to identify the most common CNV in plasma samples, followed by ddPCR verification of chosen biomarkers. CNV alterations in CDK genes were identified in 33.3% of FFPE samples (Clark IV, V only). Detection of the same genes in MM plasma showed no significance, neither compared to healthy plasmas nor between pre- versus post-surgery plasma. Sequencing data showed the most common CNV occurring in 6q27, 4p16.1, 10p15.3, 10q22.3, 13q34, 18q23, 20q11.21-q13.12 and 22q13.33. CNV in four chosen genes (*KIF25*, *E2F1*, *DIP2C* and *TFG*) were verified by ddPCR using 2 models of interpretation. Model 1 was concordant with lcWGS results in 54% of samples, for model 2 it was 46%. Although CDK genes have not been proven to be suitable CNV liquid biopsy biomarkers, lcWGS defined the most frequently affected chromosomal regions by CNV. Among chosen genes, *DIP2C* demonstrated a potential for further analysis.

## Introduction

Malignant melanoma (MM), a type of skin cancer that originates in melanocytes, is known for its aggressive nature and ability to rapidly metastasize. In addition to common genetic alterations such as *BRAFV600E* or *NRAS* mutations, copy number variations (CNV) in various genes may contribute to tumor transformation^1^.

The genes *CDKN2A/B* and *CDK4* (also referred to as *CDK* genes, in the text), frequently altered in malignant melanoma, play a critical role in tumor development and progression^2^. *CDKN2A/B* genes encode proteins that regulate the cell cycle by inhibiting cyclin-dependent kinases (CDKs), while *CDK4* promotes cell cycle progression by phosphorylating the retinoblastoma protein (pRB). Mutations or deletions in *CDKN2A/B* genes and amplification of *CDK4* are commonly observed in melanoma, contributing to uncontrolled cell proliferation and tumor growth^3–5^. Several studies have defined genes/chromosomal regions where frequent deletions or amplifications linked to MM have been detected^6–9^, however, information regarding the CNV profiles identified from circulating tumor DNA (ctDNA) of MM patients is limited.

Methods to detect these quantitative changes include fluorescent in situ hybridization (FISH), comparative genomic hybridization (CGH), microarray-based methods such as aCGH (array CGH), PCR-based methods (quantitative/digital), multiplex ligation-dependent probe amplification (MLPA), and whole-exome/whole-genome sequencing^10,11^. MLPA is a widely used technique for CNV identification^11^. Despite its cost-effectiveness and well-established use, its drawbacks include time consumption and gene-specific limitations. Droplet digital PCR (ddPCR), a simple and easy-to-interpret method, can be used for precise target DNA quantification at high levels of sensitivity and specificity^12^. Yet, challenges arise in its complexity and elevated expenses, particularly when analyzing a large number of samples or targets. Low-coverage whole-genome sequencing (lcWGS, approximately 0.5–1 × coverage) has lower accuracy or higher error rates compared to traditional high-coverage sequencing (hcWGS, approximately 30 × coverage) methods, but its advantages include lower financial costs, faster execution, and the ability to process a larger number of samples at once. LcWGS is capable of identifying various kinds of genetic aberrations, including single nucleotide polymorphisms (SNP), insertions and deletions (indels), or structural variations such as CNV^13,14^. LcWGS analysis has also been used to identify CNVs from ultra-low input concentrations of DNA^15^, suggesting that it is suitable for circulating DNA samples. Copy number changes have been examined using lcWGS from liquid biopsy samples of pediatric patients with brain tumors^16^, ovarian cancer^17^, or colorectal cancer^18^.

Several computational methods differentiating in main detection principles, segmentation, correction and normalization or necessity of using a healthy reference have been developed to detect CNV from low-coverage sequencing data, including BIC-seq2, Canvas, CNVnator, FREEC, HMMcopy, QDNAseq, cn.MOPS, CNVkit, or WisecondorX^19,20^. In our study, we used the freely accessible tool WisecondorX for CNV detection from lcWGS data. WisecondorX was created by improving its previous version Wisecondor, designed specifically for prenatal testing which allowed its wider applicability and outperformed other detection tools designed for the same purpose^20^.

The aim of this study was to assess the suitability of genes *CDKN2A*, *CDKN2B*, and *CDK4*, frequently affected in MM patients, as potential targets for detecting CNV via liquid biopsy. Subsequently, we aimed to identify the most frequently occurring CNV in plasma samples from melanoma patients and select four genes exclusively found in tumor patients, which could potentially function as diagnostic or predisposition biomarkers. These selected genes were then subjected to verification of their deletion or duplication using ddPCR.

## Methods

### Ethics approval, informed consent and data availability

The study design and conduct complied with all relevant regulations regarding the use of human study participants and was conducted in accordance with the criteria set by the Declaration of Helsinki. All subjects have provided written, informed consent to participate in the study and for the data to be published. This prospective study was approved by the University Ethics Committee, identified by the approval number EK 73/2018. The Ethics Committee at the Jessenius Faculty of Medicine, Comenius University in Martin was registered under no. IORG0004721 at the US Office for Human Research Protection, US Department of Health and Human Services and after approval, the Committee got a certification with the code "IRB00005636 Jessenius Faculty of Medicine, Comenius University in Martin IRB # 1". All methods were carried out in accordance with relevant guidelines and regulations. Some data analysed during this study are included in supplementary information files. Some generated data/datasets/raw big data files are available from the corresponding author upon reasonable request.

### Patients

In total, 60 patients with malignant melanoma diagnosis were enrolled in this study, 46 with present primary tumor (referred as primary tumor patients, 1MM-46MM, Supplementary Table 1) and 14 with present metastases of MM (post-primary tumor excision) at the time of peripheral blood withdrawal (referred as metastatic patients, 47MM-60MM, Supplementary Table 2). Primary tissue samples from 46 patients (1MM-46MM), pre-surgery plasma from all 60 patients and post-surgery plasma from 38 patients (1MM-32MM, 47MM-52MM) were collected. Post-operative plasma was obtained approximately 3 months after surgery. For MLPA analysis, 5 non-tumor formalin-fixed, paraffin embedded (FFPE) skin tissues were used as reference samples. Plasma of eight healthy participants was used in digital PCR experiments as reference. These samples were collected in cooperation with the Clinic of Dermatovenerology, Department of Plastic Surgery and Department of Pathology of Jessenius Faculty of Medicine and the University Hospital of Martin, Slovakia. Sequencing results were compared with lcWGS of circulating DNA from 127 non-cancerous plasmas collected as a part of the already existing PreveLynch dataset (NFP313010V578).

### Genomic and circulating DNA isolation and quantification

DNA from tumor and non-tumor FFPE tissue samples was isolated using the blackPREP FFPE DNA Kit (Analytik Jena, Jena, Germany), according to the manufacturer's instructions, eluted into 50 μl and stored at − 20 °C. Tumor FFPE blocks contained neoplastic tumor tissue accompanied by a thin layer of adjacent marginal tissue. Peripheral blood of the patients was collected into EDTA tubes and transported to the laboratory within 24 h of collection. From each blood sample, plasma was separated by a first centrifugation at 2200 g for 8 min (at 4 °C), followed by transfering the plasma into a new 1.5 ml tube and a second centrifugation at 20,000 g for 8 min (at 4 °C). The plasma was then stored at − 80 °C until extraction. Circulating DNA from plasma of MM patients (N = 60) and healthy participants (N = 127) was isolated using the QIAamp Blood Mini Kit (Qiagen, Hilden, Germany). The protocol for cell-free DNA (cfDNA) isolation was modified, isolation was performed from 680 μl of plasma, followed by adding 68 μl of protease and 680 μl of AL buffer. After 15 min incubation at 56 °C, 700 μl of absolute ethanol was added and this mix was subsequently plated on a QIAamp Mini spin column. Next steps were performed according to the manufacturer's instructions. Circulating DNA was eluted into 37 μl and stored at − 20 °C. Plasma samples from healthy participants (N = 8) used in ddPCR analysis were isolated using a DSP virus kit (Qiagen, Hilden, Germany), according to the manufacturer's instructions, with the fact, that the volumes were proportionally changed to the plasma volume of 1.5 ml. Circulating DNA was eluted to 20 μl and stored at − 20 °C. The Qubit dsDNA HS assay kit (Life-Technologies, Carlsbad, California, USA) and the Qubit 2.0 fluorometer (Invitrogen, Waltham, Massachusetts, USA) were used for circulating DNA quantification. For FFPE samples, the Nanodrop One (Thermo Fisher Scientific, Waltham, Massachusetts, USA) was used.

### MLPA analysis

FFPE primary tumor tissue samples (N = 46) were analyzed using the MLPA panel "SALSA® MLPA® Probemix P419-B1 CDKN2A/2B-CDK4" (MRC Holland, Amsterdam, Netherlands). This panel was used exclusively to detect deletions or duplications in the *CDKN2A*, *CDKN2B* and *CDK4* genes only. The initial concentration of both test and reference gDNA was 20 ng/μl, 5 μl (100 ng per reaction) was used. MRC Holland general MLPA protocol was followed. Mix containing HIDI formamide (9 μl) and size standard LIZ500 (0.2 μl) (Applied Biosystems, Waltham, Massachusetts, USA) was pipetted into each well in the plate. To this mix, 0.7 μl of PCR product was subsequently added and denaturation was performed at 86 °C for 3 min. After cooling, the plate was analyzed on an ABI 3500 instrument (Applied Biosystems, Waltham, Massachusetts, USA). The analysis of the results was performed in two softwares, Coffalyser.Net™ V.220513.1739, https://www.mrcholland.com/technology/software/coffalyser-net (MRC Holland, Amsterdam, Netherlands) and GeneMarker® V3.0.1., https://softgenetics.com/products/genemarker/ (Softgenetics, State College, Pennsylvania, USA). In case of equivocal results, samples were further tested and confirmed by ddPCR. The results were expressed as final ratio (FR), the ratio between the intensity of the signal from the target probe and the intensity of the signal from the reference probes. It was used to quantify whether a given segment of DNA is present in a sample in a normal amount (ratio close to 1), is amplified (FR greater than 1.3), or is deleted (FR less than 0.65).

### Droplet digital PCR

Initially, 20 μl of ddPCR reaction was prepared. This mixture contained 17 μl of mastermix, composed of 10 μl of EvaGreen Supermix, 2 μl of forward and reverse primer (Table 1, final concentration 100 nM) and 5 μl of ultrapure water. Mastermix was prepared separately for each primer set. Then, 3 μl of sample (either 3 μl of circulating DNA or 1 μl of genomic DNA filled up with ultrapure water) was added to the mastermix. In each run, a negative control, 3 μl of ultrapure water, was run with each mastermix to exclude contamination of the reagents. Final volume (20 μl) was pipetted into the middle rows of DG8™ Cartridge (Bio-Rad Laboratories, Hercules, California, USA). Then 70 μl of Droplet Generation Oil for EvaGreen was applied to the bottom wells. The DG8™ Cartridge was then placed in the QX200™ Droplet Generator (Bio-Rad Laboratories, Hercules, California, USA) and 40 μl of generated droplets were pipetted from the upper wells into the 96-well plate. The PCR plate was covered with a pierceable foil and heat-sealed using Bio-Rad PX1™ (Bio-Rad Laboratories, Hercules, California, USA). The PCR plate was then placed in a T100™ thermal cycler (Bio-Rad Laboratories, Hercules, California, USA) and protocol was initiated by denaturation (95 °C for 10 min) followed by 40 cycles of denaturation (95 °C for 30 s) and annealing (59 °C for 1 min) followed by signal stabilization (4 °C for 5 min, 90 °C for 5 min). After PCR amplification, the 96-well plate was transferred to a QX200TM Droplet Reader (Bio-Rad Laboratories, Hercules, California, USA). Data obtained from the QX200™ were analyzed and interpreted with QuantaSoft V.1.7 software (Bio-Rad Laboratories, Hercules, California, USA). Droplets were divided into two clusters (positive, negative/empty) according to the fluorescence emission analysis of the intercalating dye. The threshold was set at the highest possible amplitude to eliminate nonspecific binding. The measured values were normalized to the value of the *SPAST* reference gene. The results were expressed as the final ratio (FR) to the *SPAST* gene, which represented a value of 1. For the genes *CDKN2A/B* and *CDK4*, we compared FR of malignant plasma to healthy plasma, no other interpretation was used. For *E2F1*, *TFG*, *KIF25* and *DIP2C* genes two models of result interpretation were used and compared. Model 1: Deletion < 0.9 and 1.1 < Amplification and model 2: Deletion < 0.65 and 1.3 < Amplification. All plasma samples (pre-surgery, post-surgery) were included in ddPCR analysis of *CDK* genes; only selected samples (based on sequencing results) we tested for *E2F1*, *TFG*, *KIF25* and *DIP2C* genes.

**Table 1:** Primer sequences of genes detected by ddPCR.

Gene	F/R	Sequence	Tm (°C)
*SPAST*	F	TTTTCCAAGTCACAAACGGACG	55.5
	R	ATTGCGGCATGCCAAGTTAG	56.1
*CDKN2A*	F	GTGCGCAGGGCTCAGA	58.9
	R	CCACCCGGACCTCCAAG	58.9
*CDKN2B*	F	CGCATGCGTCCTAGCATC	56.8
	R	GGGGAAGCCTGCCCAA	58.9
*CDK4*	F	TTCCCATCAGCACAGTTCGT	56.4
	R	TCCAGTCGCCTCAGTAAAGC	57
*E2F1*	F	GCTACCGGGAACGATTCCAT	57.2
	R	AGGCCTGGAGTTGTGTTGAA	56.4
*TFG*	F	GGCGCCTACGGGAATAAGTT	57.5
	R	CCAAGGAACTAGGCTCCGAT	56.4
*KIF25*	F	ATAGAGACCAGACGGAGGCT	57.1
	R	ACCAAGTGGGTGTCATAGGC	57.2
*DIP2C*	F	AGGCTTACTGCTGACTGCAT	56.2
	R	TATGTTGTGGGCAGCGTCTT	56.7

### Low coverage whole genome sequencing

Pre- and post-surgery plasma samples of primary tumor patients (N = 46) were included in whole genome sequencing analysis. Illumina TruSeq Nano DNA Library Prep kit (Illumina, San Diego, California, USA) was used for DNA library preparation. The volumes of reagents used were optimized for processing low-concentration samples. The DNA fragmentation step was omitted as this was fragmented circulating DNA. "End Repair Mix", which converts damaged or incompatible 5'- and 3'-ends to DNA with blunt ends, was used to modify the ends of the fragments. After end repair, no selection of fragments by size was done using SPB (Sample Purification Beads) purification magnetic beads, but a fourfold volume of beads compared to the sample volume was used to pick up nucleic acids, regardless of their size due to the lack of input material. Next steps, adenylation of 3' ends and ligation of adapters were performed according to manufacturer’s instructions. Subsequently, PCR was used to selectively enrich those DNA fragments that had adapter molecules at both ends and to amplify the amount of DNA in the library. The amplifications were performed using PCR, with initial denaturation (95 °C, 3 min.), followed by 8 cycles of denaturation (98 °C for 20 s.), annealing (60 °C for 15 s.) and extension (72 °C for 30 s), finished with final elongation at 72 °C for 5 min. Quality control and library size distributions were performed on the Agilent 2100 Bioanalyzer system, using the Agilent High Sensitivity DNA Kit (Agilent Technologies, Santa Clara, CA, USA). A low coverage whole genome sequencing approach (~ 1 × Genome) was used to digitize the prepared DNA libraries. Illumina NextSeq 2000 P3 v3 sequencing kit (200 cycles) (Illumina, San Diego, California, USA) was used for WGS using paired-end reads, 2 × 100 bp in length on the Illumina NextSeq 2000 platform.

### Biostatistical and bioinformatical analysis

Digitized fragments obtained by whole genome sequencing with quality of bases lower than 20 were trimmed by the trimmomatic tool. Reads longer than 35 bp were mapped to reference genome GRCh38/hg38 using the BWA tool^21^. In order to detect sample’s CNV, we applied WisecondorX on mapped .bam files. Main steps of WisecondorX detection process include: imaginary dividing genome into non-overlapping segments of the same length, bins (in our case 20 kbp) and expressing the depth of coverage of the reads for each bin; creating reference from non-cancerous samples from approximately half of the control samples with differences in gender, age and diseases of non-cancerous origin with intrasample normalization and filtering non-informative regions (regions with no reads, reads with bad mapping quality, or regions with high variance in coverage) and applying circular binary segmentation on z-score calculated from normalized and filtered bins for detection of CNVs as a significant deviation from non-cancerous reference.

Digital PCR data expressed as final ratio were explored and analyzed in R, ver. 4.0.5. Distribution of the Final Ratio (FR) was visualized by boxplot overlaid with swarmplot, density plot and Quantile–Quantile plot with the 95% confidence band constructed by bootstrap. Change of FR between pre- and post-surgery stages was visualized by the spaghetti plot. Linear mixed model of the form Fr ~ group + surgery + (1|id) was fitted to the data by REML method. There, group was a factor with three levels: control, primary, metastasis and surgery was a factor with two levels: pre, post. The fitted model was subjected to diagnostic analysis of residuals. If outliers were detected (two outlier for *CDK4*, none for *CDKN2A* and *CDKN2B*) then they were excluded from considerations and the model was refit and subjected to diagnostics. Effect size was quantified by the Adjusted R^2. The fitted linear mixed model was used to estimate the marginal means. Post-hoc pairwise comparisons were performed with the Benjamini-Hochbergh correction of p-values in the case of the comparisons for group factor. The marginal means together with their 95% confidence interval were visualized by the marginal means plot (Supplementary material 1).

## Results

### CNV in the *CDK4*, *CDKN2A* and *CDKN2B* genes present only at later stages of the disease

We initially identified the presence of CNV in the *CDK4*, *CDKN2A* and *CDKN2B* genes in tissue FFPE samples from patients with primary tumors (N = 46), using MLPA analysis. FFPE sample MM39 could not be analyzed neither by MLPA nor ddPCR, due to poor quality. We detected CNV changes in at least one gene (*CDKN2A*, *CDKN2B*, *CDK4*) in 33.3% of samples (15 of 45) (Supplementary Table 3). We only detected CNV changes in Clark IV and V, not in the lower stages. The percentages of tissue samples with CNV were 0% (0 of 6), 0% (0 of 7), 48.1% (13 of 27), and 40% (2 of 5) for stages II, III, IV, and V, respectively. The *CDKN2A* gene was deleted in 11 samples (*BRAF* WT (N = 9), *BRAFV600E* (N = 2)), the *CDKN2B* gene in seven (*BRAF* WT (N = 5), *BRAFV600E* (N = 2)). The *CDK4* gene was amplified in 4 samples (*BRAF* WT (N = 1), *BRAFV600E* (N = 1), N/A (N = 2)), and in 1 of these, *CDK4* amplification was accompanied by amplification of *CDKN2A* and *CDKN2B.* No correlation between specific tumor type, location or *BRAF* status was observed.

### Quantification of total circulating DNA from patients' blood and correlation with stage and type of malignant melanoma

**Figure 1:**
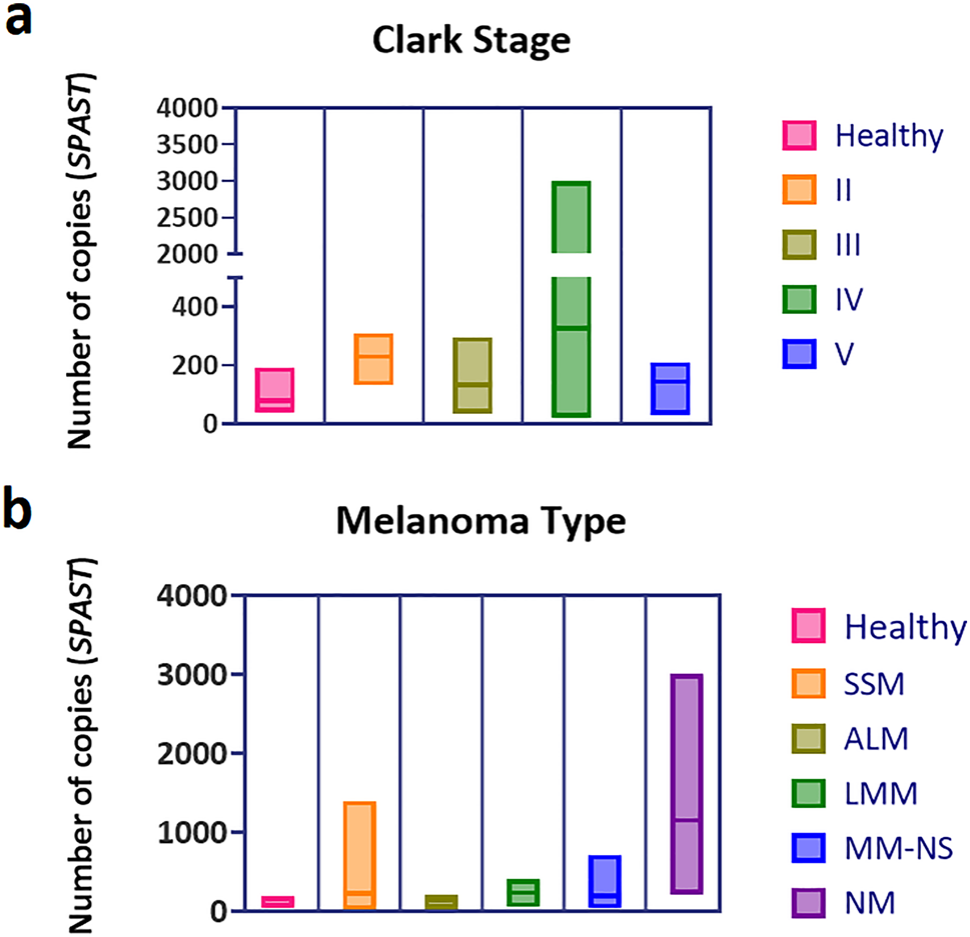
Quantification of total circulating DNA from patient blood using ddPCR and the *SPAST* reference gene. Correlation with Clark stage (**a**) and MM type (**b**). Abbreviations: SSM = superficial spreading melanoma, NM = nodular melanoma, LMM = lentigo maligna melanoma, ALM = acral lentiginous melanoma.

Total circulating DNA in the blood of patients was quantified using the *SPAST* reference gene and digital PCR. The mean copy number of the *SPAST* gene was 78 (38–193 copies) in healthy participants, 229 (Clark II; 131-310 copies), 134 (Clark III; 34–296 copies), 327 (Clark IV; 22-3000 copies) and 145 (Clark V; 30-210 copies) (Fig. 1a) and thus averaged a 2.9- (Clark II); 1.7- (Clark III); 4.2- (Clark IV) and 1.9-fold (Clark V) increase in circulating DNA compared to healthy participants. In terms of tumor type, the mean number of copies was 229 for superficial spreading melanoma (SSM; 22-1,396 copies), 133 for acral lentiginous melanoma (ALM; 30-210 copies), 231 for lentigo maligna melanoma (LMM; 55-406 copies), 188 for MM-NS (Non-Specific) (MM-NS; 34-714 copies) and 1151 copies for nodular melanoma (NM; 208-3000 copies) (Fig. 1b).

### The copy number of *CDK* genes in the plasma of patients is not significantly different from the plasma of healthy participants

**Figure 2:**
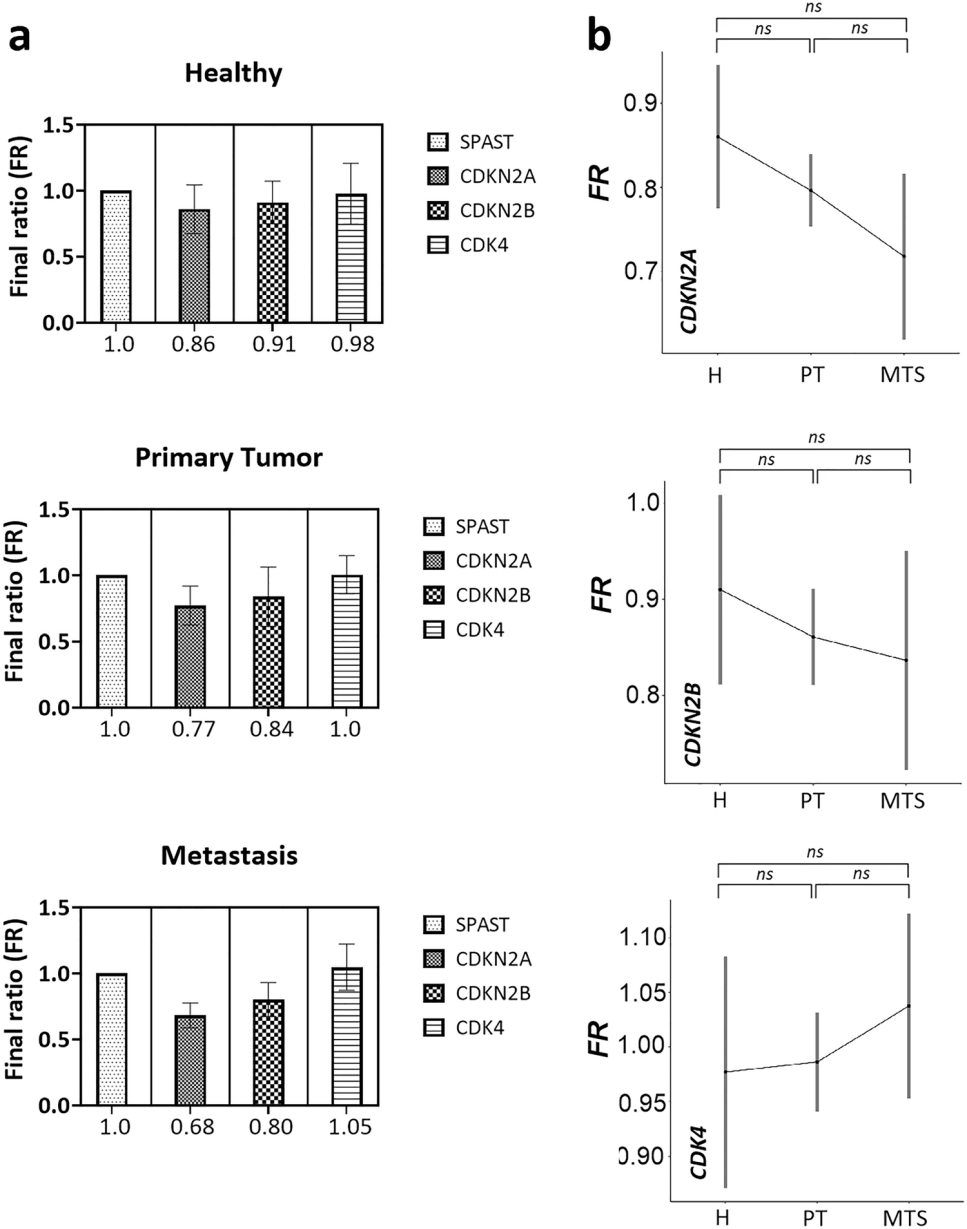
Comparing FR in genes *SPAST*, *CDKN2A*, *CDKN2B* and *CDK4* from plasma samples of patients and healthy participants. (**a**) FR of *SPAST* and *CDK* genes detected in healthy and tumor plasma; (**b**) comparison of FR for *CDKN2A*, *CDKN2B* and *CDK4* genes between healthy participants and patients with primary tumor and metastasis. *Abbreviations: FR* = *final ratio, Z* = *healthy, PN* = *primary tumor, MTS* = *metastasis.*

We subsequently detected CNVs in the *CDK4*, *CDKN2A*, and *CDKN2B* genes from plasma of MM patients (N = 60), including 46 patients with present primary tumor and 14 patients with present metastases, at the time of peripheral blood collection. The results were compared with healthy plasma (N = 8). For the *CDKN2A* gene, the mean final ratio (FR = final ratio), compared with healthy plasma, was 0.09 and 0.18 lower in patients with primary tumor and metastatic patients, respectively. For the *CDKN2B* gene, this was a mean reduction in FR of 0.07 and 0.11 in patients with primary tumor and metastatic patients, respectively. The *CDK4* gene was on average increased by 0.02 and 0.07 in primary tumor and metastatic patients, respectively, in terms of FR (Fig. 2a). Statistically, there was no significant change between healthy plasmas and plasmas from primary tumor patients (*CDKN2A,* *p* = 0.186; *CDKN2B,* *p* = 0.563; *CDK4,* *p* = 0.874); or plasmas from metastatic patients (*CDKN2A,* *p* = 0.100; *CDKN2B,* *p* = 0.563; *CDK4,* *p* = 0.564). There was also no significant difference between primary tumor patients and metastatic patients (*CDKN2A,* *p* = 0.186; *CDKN2B,* *p* = 0.695; *CDK4,* *p* = 0.564) (Fig. 2b).

### The copy number of *CDK* genes in pre-surgery plasma is not significantly different from post-surgery plasma

**Figure 3:**
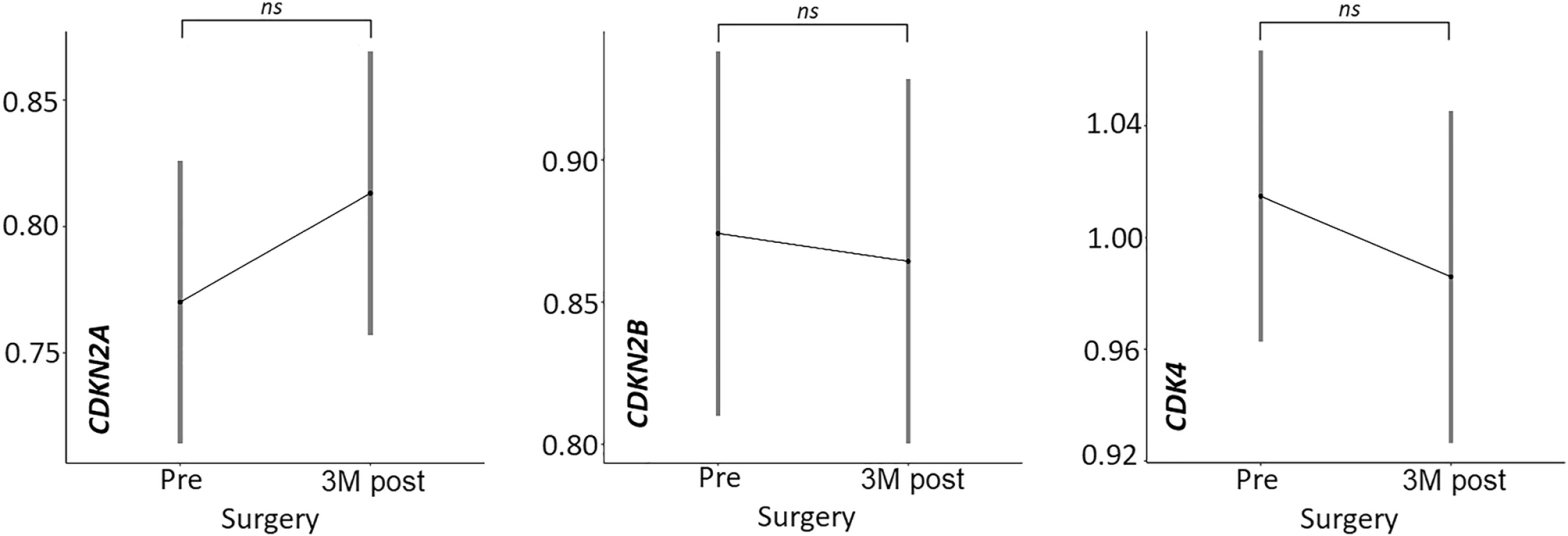
Comparison of CNVs in *CDKN2A*, *CDKN2B* and *CDK4* genes in pre- and 3 M post-surgery (collected approximately 3 months after tumor removal) plasma from patients with primary tumor and metastasis.

From 38 patients (N = 32 primary tumor; N = 6 metastatic) we also had post-surgery plasma, collected on average 3 months (approximately 97 days) after the primary tumor or metastasis was removed. For *CDKN2A*, *CDKN2B* and *CDK4* genes, the differences in final FR ratio between pre-surgery and post-surgery plasma were 0.05 (↑); 0.01 (↓) and 0.05 (↓), respectively. Statistically, there was no significant change between pre- and post-operative plasma (*CDKN2A,* *p* = 0.206; *CDKN2B,* *p* = 0.793; *CDK4,* *p* = 0.337) (Fig. 3).

### The most frequently occurring CNVs in MM plasma

Genome-wide lcWGS analysis of primary tumor patient’s plasma samples (N = 46) (collected both pre- and post-surgery) showed different copy number alterations. By filtering out CNVs that were present in at least 4 patients and in no healthy participants, we obtained 47 chromosomal regions encompassing a total of 1813 protein-coding genes (Supplementary Material 2), interestingly not containing *CDKN2A*, *CDKN2B* and *CDK4* genes, despite being detected in 33.3% of tissue samples. In total twelve patients respected the selected criteria (3,5,10,16,18,19,30,34,38,39,43-MM). Eleven of these samples were classified as stage IV, however, the tumor types and ICD classifications varied, with no single category predominating. The cytogenetic bands in which CNV were most frequently observed were 6q27 (N = 7), 4p16.1, 10p15.3, 10q22.3, 13q34, 18q23, 20q11.21-q13.12 and 22q13.33 (N = 5), 3q12.2, 5p15.33, 6q25.3-q27 (N = 4), and others (Supplementary Table 4; Supplementary Material 2). These CNVs were mostly localized in or around the telomeric or centromeric portion of the chromosome. Of the 47 chromosomal locations filtered, 26 contained at least one COSMIC gene. The average CNV length at these 47 sites was 3,509,807 nucleotides (ranging from 7073 to 24,483,676) (Supplementary Table 4). The ratio of amplifications and deletions was 33%:67%. The majority of detected filtered-CNVs were from preoperative samples from patients 19MM, 30MM, 34MM, and 38MM and postoperative plasma from patient 18MM. Deletions and amplifications from these five samples accounted for up to 67.4% of the total CNVs. Four of these five specimens share Clark stage (IV), and three specimens share tumor location, on the upper extremity (C43.6). Three of these five cases were superficial spreading melanoma (SSM).

### Verification of results by ddPCR

We chose 4 genes, *KIF25*, *E2F1*, *DIP2C* and *TFG,* to verify the lcWGS results by ddPCR. These genes were cancer-associated^22–25^, specifically found in tumor samples only and were absent in healthy participants (Supplementary Material 2). Additionally, these genes were chosen because they were also present outside the five samples (18MM, 19MM, 30MM, 34MM, 38MM) and have the potential to serve as therapeutic targets^22,23^ or diagnostic and predisposing biomarkers^24,25^ CNVs of *KIF25* gene were detected in 8 patients, in case of *E2F1* and *DIP2C,* in 6 patients and *TFG* in 4 patients (out of 46 patients). Using ddPCR we analyzed tumor gDNA and ctDNA of patients, in whom CNVs in these genes were detected by lcWGS. We used two scoring models to analyze the ddPCR results: Model 1: Deletion < 0.9 and 1.1 < Amplification and Model 2: Deletion < 0.65 and 1.3 < Amplification. We adopted model 2 from the MLPA analysis and proposed the more benevolent model 1 for comparison. When comparing ddPCR results of pre- and post-operative plasma with NGS results (Deletion/Amplification/No CNV), the results evaluated by model 1 were concordant with NGS results in 54% of samples (20 of 37); for model 2, this was a 46% concordance (17 of 37). Looking at each gene separately, comparing lcWGS and ddPCR (model 1), consistent results between these two methods were in 5 of 12 samples for *KIF25*, for *E2F1* 6/9, for *DIP2C* 6/10 and for *TFG* 3/6 samples. We also compared the preoperative plasma results (lcWGS, ddPCR) with those of FFPE tissue evaluated by model 1 and 2 (ddPCR). FFPE tissue (model 1) vs. NGS plasma, plasma-model 1 and plasma-model 2, showed 42% (10 of 24), 33% (8 of 24), and 46% (11 of 24) similarity, respectively. For tissue (model 2), it was 29% (7 of 24), 29% (7 of 24), and 63% (15 of 24), respectively (Table 2).

**Table 2:**
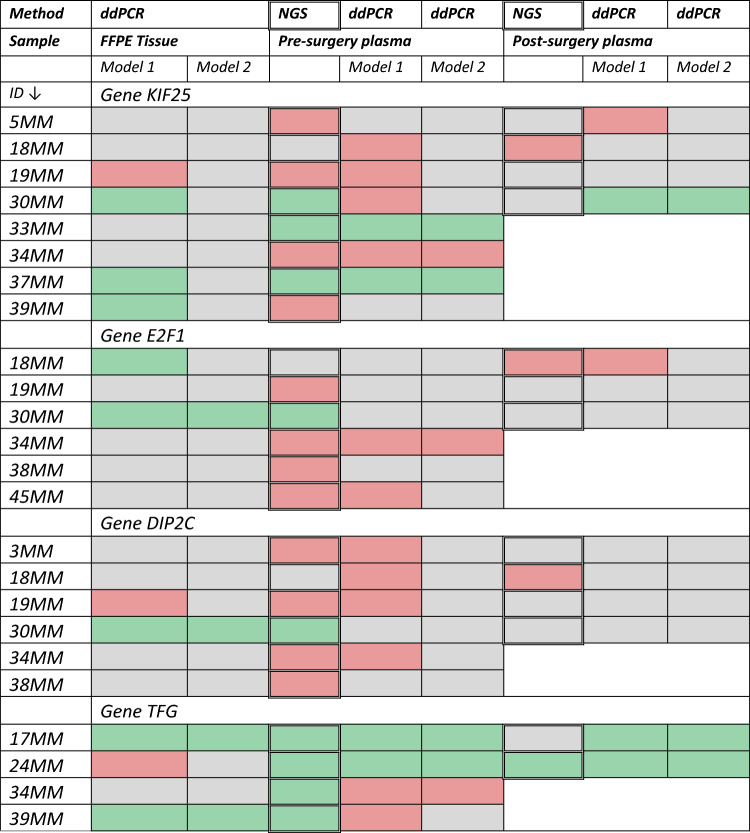
Comparison of NGS and ddPCR results, plasma and tissue. For ddPCR, two scoring models were used. Grey = no CNV, green = amplification, red = deletion.

## Discussion

In malignant melanoma, CNVs are associated with tumor development and progression. Melanomas with a poor prognosis show significantly more frequent genetic abnormalities and a significantly higher number of CNVs have been reported compared to melanoma cases with a good prognosis^26^.

At the beginning, we looked at copy number changes in genes *CDK4*, *CDKN2A* and *CDKN2B*. Although primarily associated with familial melanoma, CNVs in these genes have been observed in all melanoma types^6–9,27–35^. Our results (on FFPE tissue samples) showed that copy number changes in these genes were present only at later stages of the disease (Clark stage IV and V), although Rákosy and colleagues^30^ showed that deletion of the 9p21 region (*CDKN2A/B*) is also present in early stages of melanoma. This circumstance could stem from the smaller sample size obtained from patients in stages II and III. It could be due to rapid tumor growth and progression^36^ or late diagnosis of the disease. Xavier et al. (2016)^37^ investigated the reasons for the delay in the diagnosis of MM. The main component of the delay was related to the patient. Only 31.3% of patients knew that melanoma was a serious skin tumor and most thought that the pigmented lesion was not important. In the *CDK4* gene, we detected amplification only. For *CDKN2A* and *CDKN2B* genes, we primarily detected deletion, but in one sample (MM30), we observed amplification. Although the *CDKN2A* gene is predominantly deleted in MM, amplification can also occur^30^. In neither case, we did not detect a combination of *CDKN2A/B* deletion and *CD4* amplification in the tissue sample, confirming that these two phenomena are mutually exclusive^38^.

Although several studies have identified copy number changes of *CDK* genes in tissue samples^39–41^, there is limited information on their capture from the plasma of MM patients. Thus, we decided to analyze CNVs in these genes in circulating ctDNA from MM patients and determine whether these aberrations may serve as a suitable biomarker for the noninvasive diagnosis of melanoma patients.

The measured values were compared with the *SPAST* reference gene. Using this gene, we were also able to quantify the total circulating DNA in the blood of the patients. Compared to healthy plasma, we detected a 2.9- (Clask II); 1.7- (Clark III); 4.2- (Clark IV); and 1.9-fold (Clark V) increase in circulating DNA in the tumor plasma. Although such quantification cannot define the ratio of cfDNA to ctDNA, it can indicate increased levels of circulating DNA in the blood. These can be elevated in various physiological and clinical conditions in addition to cancer, such as myocardial infarction^42^, trauma^43^, during pregnancy^44,45^, or in post-transplant patients derived from a donor organ^46,47^. In cancer, higher levels of circulating DNA (cfDNA) are associated with a greater disease burden, more advanced disease stage and a higher number of metastatic sites^48^. Measurement of circulating DNA levels has potential use in response to immunotherapy. Rising levels of DNA from tumor cells targeted by immunotherapy may indicate treatment success^49^.

Copy number analysis of *CDK* genes from the plasma of the patients did show deletion in the *CDKN2A/B* genes and amplification in the *CDK4* gene compared to healthy samples, but these changes were not statistically significant. We also found no significant difference between pre-operative and post-operative plasma, which was collected approximately 3 months after tumor removal, ensuring that elevated levels of circulating DNA due to the surgical procedure were not present. Thus, we concluded that these CNV biomarkers are not suitable for liquid biopsy and decided to identify new ones that would be more suitable for non-invasive diagnosis from blood. For this purpose, we chose low-coverage whole-genome sequencing (lcWGS).

When comparing several methods, it is WisecondorX that has produced the best results in terms of variance, distributional assumptions and basic ability to detect true variation^20^.

Various genome-wide studies have defined genes/chromosomal regions in which frequent deletions/amplifications associated with MM have been observed^6–9^, but information regarding the CNV profile of ctDNA isolated from MM patients is limited. Therefore, we set out to identify the most common copy number changes from plasma samples of MM patients. In previous studies, frequent CNV changes in MM tissue samples were located, for example, on chromosomes 7q, 4q, 8q, 6p, 6q, 10q, 11q, 12q, 20q, 22q^6,29,33,50^. Our results pointed to the most affected chromosomal regions (whether deletions or amplifications) 6q, 4p, 13q, 18q, 20q and chromosomes 10, 15, 16, 17, 20 or 22, which partially corresponds with the observed results presented in the previous literature. Although CNVs were detected in a high number of protein coding genes, when filtering the genes present in patients only (4 and more), we reduced it to 47 chromosomal regions mostly located in or around the telomeric or centromeric regions. Furthermore, CNVs in *CDKN2A*, *CDKN2B*, and *CDK4* were not in the filtered gene set, despite their presence in one-third of the tissue samples. This discrepancy indicates a potentially low concordance between tissue and plasma samples, underscoring the necessity for computational tools like WisecondorX, when analyzing liquid biopsy samples. Such programs are essential for generating reliable CNV references from non-cancerous samples, enabling the precise identification of significant CNV alterations in cancer plasma.

In general, chromosomal breaks and rearrangements are more frequent near centromeres and telomeres, contributing to the formation of CNVs, also, these regions play crucial roles in maintaining genomic stability and regulating gene expression. Alterations in these regions can disrupt essential functions, potentially promoting cancer development^51,52^. On the other hand, centromeres and telomeres contain highly repeated sequences, making them challenging for accurate mapping and alignment of short-read sequencing technologies resulting in possible false positive results when using shallow sequencing methods^53^. That is why we decided to verify the lcWGS results using ddPCR. We chose 4 genes (*KIF25*, *E2F1*, *DIP2C*, *TFG*) that were present in at least 4 tumor patients, were not present in the healthy population, were detected outside of patients 18, 19, 30, 34, and 38, and have the potential to serve as therapeutic targets^22,23^ or diagnostic and predisposing biomarkers^24,25^. When searching these genes in the GDC (Genomic Data Commons) data portal of National Cancer Institute^54^, in project ID: TCGA-SKCM where they identified CNV in 467 melanoma samples, deletion predominated and was detected in 50.54% of samples for gene *KIF25* and in 48.39% samples for gene *DIP2C*. Predominantly, gain of gene *E2F1* was detected in 38.54% samples and in case of *TFG*, gain was captured in 13.92% and loss in 14.56% of melanoma samples.

KIF25 is one of the members of the kinesin-like protein family, which are microtubule-dependent molecular motors and thus play an important role in the processes of intracellular transport and cell division. The *KIF25* gene (Cytoband: 6q27) has been associated with various tumor types, including breast cancer^22^, osteosarcoma^55^, and malignant mesothelioma^56^. Groth-Pedersen et alia^55^ showed that inhibition of *KIF25* expression in human osteosarcoma cells by U2OS significantly reduced their proliferation. In breast cancer, Zou and colleagues^22^ found out that estrogen induces the expression of *KIF* genes, including *KIF25*, and they also pointed out the possible deregulation of the kinase family by the ANCCA (AAA nuclear coregulator cancer associated) coregulator. Although in the TCGA database^54^ it was shown that deletion of this gene was predominant, other studies show that the overexpression of this gene^22,55^ can cause cell proliferation. That means that we can assume that amplification that was detected by lcWGS in three of our MM samples, may cause increased proliferation of tumor cells, in contrast, deletion detected in four samples, may serve as a cell defense mechanism against neoplastic-driven proliferation.

E2F1 is a transcriptional activator that plays a major role in cell cycle control under physiological and pathological conditions. E2F1 protein acts in cooperation with pRB and CDKs^57^. CNV alterations in the *E2F1* gene (Cytoband: 20q11) or its increased expression have been localized in several tumor types, renal cell carcinoma^58^, prostate cancer^59^ or hepatocellular carcinoma^60^. Amplification of the *E2F1* gene has been observed in melanoma cell lines (compared to normal melanocytes) and also in melanoma metastases localized in lymph nodes. In addition, Western blot analysis demonstrated increased levels of E2F1 protein in 8 of 9 melanoma cell lines compared to normal melanocytes^61^. In our samples, amplification was detected in only 1 sample, with predominant deletion in 5 ctDNA samples. This does not correlate neither with results from other studies nor with TCGA database^54,59–61^. This gene may serve as another potential therapeutic target. Inhibition of the E2F1 protein using the small molecule inhibitor HLM006474 can induce cell death in melanocytic tumor cells resistant to BRAF inhibitors^23^. Moreover, CNV alterations in this gene may serve as a predisposing biomarker for skin cancer^25^.

The *DIP2C* gene, a member of the disco-interacting protein homolog 2 family, is highly expressed in the brain and influences its development and function^62^. A global transcriptome study in DIP2B-deficient mice (DIP2B is a paralog of DIP2C) suggested that this gene plays multiple roles in cell proliferation, migration, and apoptosis^63^. Loss of *DIP2C* can affect DNA methylation and changes in gene expression, cellular senescence, and epithelial-mesenchymal transition in cancer cells^64^. Aberrations of the *DIP2C* gene (Cytoband: 10p15.3) have been detected in breast cancer^24^, prostate cancer^65^ or spitzoid melanoma^66^. Liu and colleagues (2023)^65^ found that exosomal miR-375 (tumor-derived) specifically targets the *DIP2C* gene, thereby regulating the Wnt signaling pathway and promoting osteoblastic metastasis and prostate cancer progression. The deletion of this gene, which was detected by lcWGS in five (5/6) of our melanoma samples, confirms the results from the studies of Li et al. (2017)^24^ and Liu et al. (2023)^65^ which showed a decrease in the expression of this particular gene in tumor cells, also our results are consistent with TCGA (TCGA-SKCM) database^54^ where deletion was detected in 48.39% of melanoma samples. Interestingly, in the study by Larson and colleagues^64^ gene expression profiling revealed 780 genes for which expression levels were affected by loss of *DIP2C*, including the *CDKN2A* gene encoding a tumor suppressor that is frequently deleted in MM.

The Trk-fusion gene (*TFG*) was amplified in four melanoma patient plasma samples according to our lcWGS results, but digital PCR also indicated a possible deletion. Although the function of this gene is currently poorly characterized, it is thought to be involved in the NF-kB and MAPK signaling pathways^67^. One of the key links of *TFG* to cancer is through fusions of this gene with others. Such fusions can generate new proteins, oncoproteins, which can interfere with normal cell cycle regulation and lead to neoplastic transformation of the cell^68–72^. Endoh et al. (2012)^73^ showed that the expression level of TFG in prostate tumor tissues was higher than in non-tumor tissues in 63.9% of cases. Targeted inhibition of *TFG* by specific silencing RNAs led to reduced proliferation of PC3 tumor cells and induction of premature senescence. The increased expression correlated with our results, which indicated amplification of this gene. As *TFG* is primarily involved in the NF-kB (important in the inhibition of apoptosis and treatment resistance in melanomas) and MAPK (activation of this signaling pathway occurs in approximately 70% of melanomas) pathways, aberrations in this gene may be considered important in the tumorigenesis of melanomas.

Overall, in the context of individual gene examination, result consistency between lcWGS and ddPCR and cross-referencing with existing studies and TCGA database, *DIP2C* demonstrated a potential for further analysis as non-invasive CNV biomarker of malignant melanoma.

When comparing lcWGS and ddPCR, the results from ddPCR were identical to those from lcWGS in 54% for evaluation model 1 and in 46% for evaluation model 2. We therefore propose to use model 1, due to the higher similarity with the NGS results, since the NGS analysis compared the results with the overall genetic profiles and in case of ddPCR, only one reference gene was used. Comparison of plasma vs. FFPE tissue (both methods, both scoring models) showed similarity ranging from 29% up to 63%, although the comparison of tissue vs. liquid biopsy samples, especially for quantitative changes, may yield discrepancies due to variations in sample types, collection methods, differences in the stability of genetic material, and the presence of cfDNA from non-tumor cells in liquid biopsy samples. The low percentage of similarity of results may be due to several limitations of both methods.

Biological limitations of lcWGS are caused by naturally highly fragmented genomes of oncological patients^18^, which produce short sequenced reads for mapping to the reference genome. Modeling of copy number profile is further complicated by a low tumor fraction, proportion of ctDNA in the total cfDNA of tumor patients, especially in those at a lower stage of the disease or in patients with slow-growing tumors^74^. The presence of cfDNA from normal cells, such as hematopoietic or endothelial cells, introduces background noise, hindering the detection and quantification of tumor-specific copy number alterations and potentially resulting in biased representations of the tumor's CNV profile. More technical limitation of CNVs is selected bin size associated with the accuracy of CNVs length and positions in general which can detect only approximate borders of CNVs rounded up to selected bin size. In other words, we can say inside which bins there are CNVs, but not exactly where inside the bin. In addition, when we overlap these positions with gene locations on the genome, it can produce false matches and it would be appropriate also to consider the length of overlapping with genes. Use of WisecondorX is also highly dependent on the selection of healthy reference samples. The more samples we use with health status in mind, the better we can model a healthy population and filter out CNVs correlated with non-cancerous diseases in the population.

The limitations of ddPCR in this case represent the introduction and optimization of the ddPCR CNV assays themselves. For liquid biopsy samples, they can present challenges, mainly due to the selection of an adequate reference gene(s) to which the copy number of the target gene will be compared. Although *SPAST* was shown to be the most suitable reference gene from tumor tissue MM samples and healthy skin samples (based on our MLPA results; data not shown) and was used as the only reference gene in non-invasive cfDNA analysis, inconsistent results when compared with lcWGS results suggest that in case of detection CNVs from circulating DNA, the inclusion of multiple reference genes would help to obtain a better and more balanced reference value. Considering the importance of CNVs in human genetic diversity and their association with multiple complex disorders^75^, it is critical to pay great attention to the selection of multiple reference genes for ddPCR analysis. Ideally, these reference genes should have a single copy in the genome and their copy number should not vary between healthy individuals and cancer patients. For example, Ma et al. (2023)^76^ identified *AGO1*, *AP3B1*, *MKL2*, and *RPP30* as suitable reference genes for CNV analysis by ddPCR because their copy numbers were not altered in either tumor or non-tumor samples. However, this research was only about genomic DNA, not circulating DNA. Another helpful point would be to determine the ctDNA fraction from the patient's total cfDNA. To do so, genetic or epigenetic profiling of tumors using next-generation sequencing is recommended^77–80^. Another challenge is the limited sample volume, which does not allow us to analyze a large number of genes simultaneously. Therefore, a more effective alternative could be the use of ddPCR with TaqMan probes, which enables multiplex analysis. The use of multiple reference genes could improve the accuracy of CNV detection by reducing the influence of variations in individual reference genes^81^.

Both methods, lcWGS and ddPCR, have their own advantages and limitations in CNV detection. Therefore, the combination of these methods appears to be beneficial for the identification of new diagnostic biomarkers and the subsequent creation of dPCR assays that can be used for early detection of the disease, monitoring its course or response to treatment. Thus, such an integrative approach provides more comprehensive information for potential use in the non-invasive diagnosis of CNV tumor changes.

### Supplementary Information

Supplementary Information 1.

Supplementary Information 2.

Supplementary Tables.
