# Supplementary material for: Novel liquid biopsy CNV biomarkers in malignant melanoma
Source: Sci Rep. 2024 Jul 9;14:15786. doi: 10.1038/s41598-024-65928-y (PMC11233564; doi:10.1038/s41598-024-65928-y)
Supplement: Supplementary file 1 — Supplementary Information 1. [file 41598_2024_65928_MOESM1_ESM.pdf]

report_CDKN2A__Oct9_2023.R

mg

2023-10-09

```
#
#
# Oct 9 2023 Mo -- CDKN2A
# Oct 5 2023 Th -- triplot; spaghetti; lin mixed model - dg, outlier,
#                  emmeans, custom contrasts
# Oct 4 2023 We -- CDK4 ddPCR CNV
#
#
# Eva Lukacova
#
```

CDKN2A

EDA

Triplot (for FR by surgery and group)

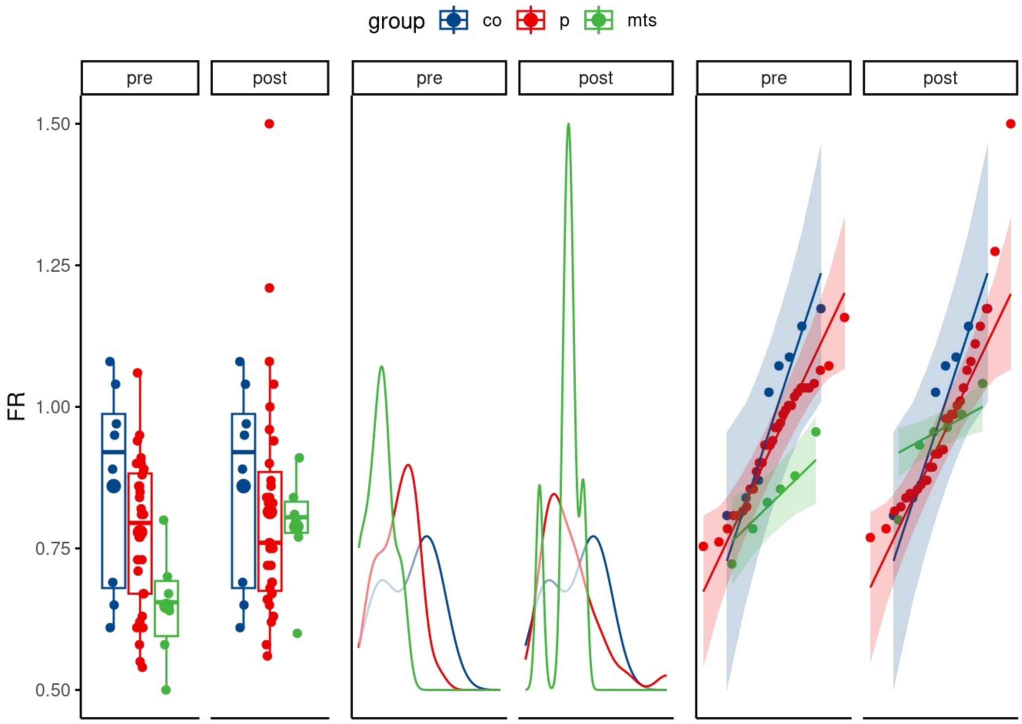

Spaghetti plot for surgery (w group wo co)

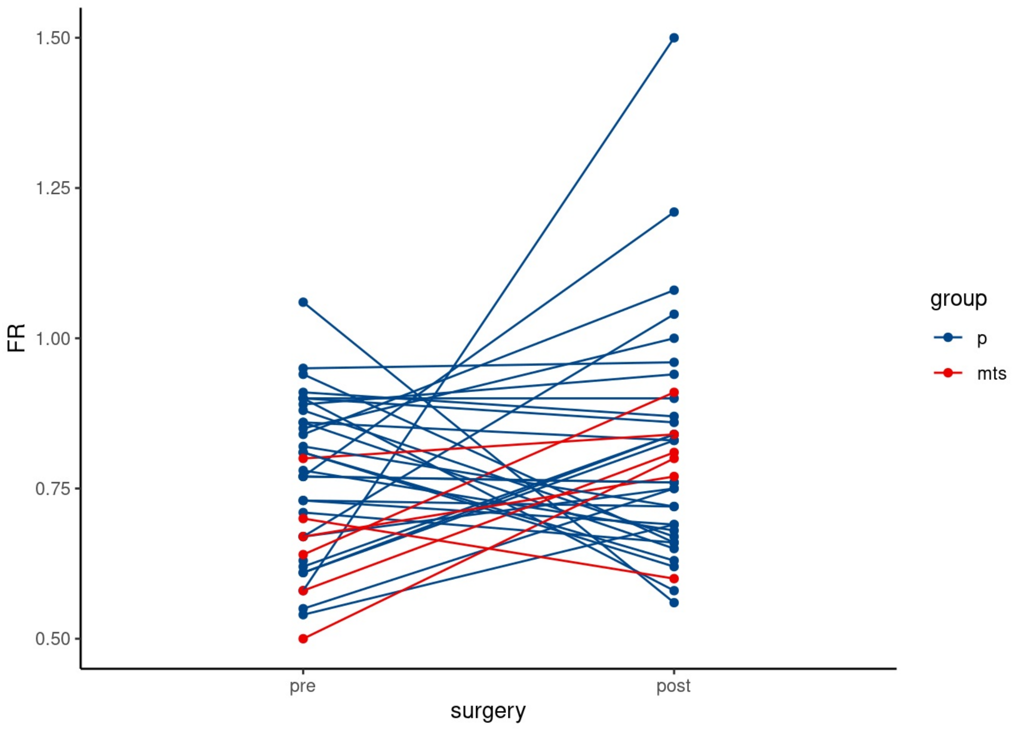

Linear mixed model (to fit Repeated measures ANOVA)

Model fit

	FR		
Predictors	*Estimates*	*CI*	*p*
(Intercept)	0.84	0.75 – 0.93	**<0.001**
group [p]	-0.06	-0.16 – 0.03	0.183
group [mts]	-0.14	-0.27 – -0.01	**0.031**
surgery [post]	0.04	-0.02 – 0.11	0.203
**Random Effects**			
σ^2	0.03		
$\tau_{00\ id}$	0.00		
ICC	0.05		
N_{id}	46		
Observations	91		
Marginal R^2 / Conditional R^2	0.069 / 0.118		

Diagnostics

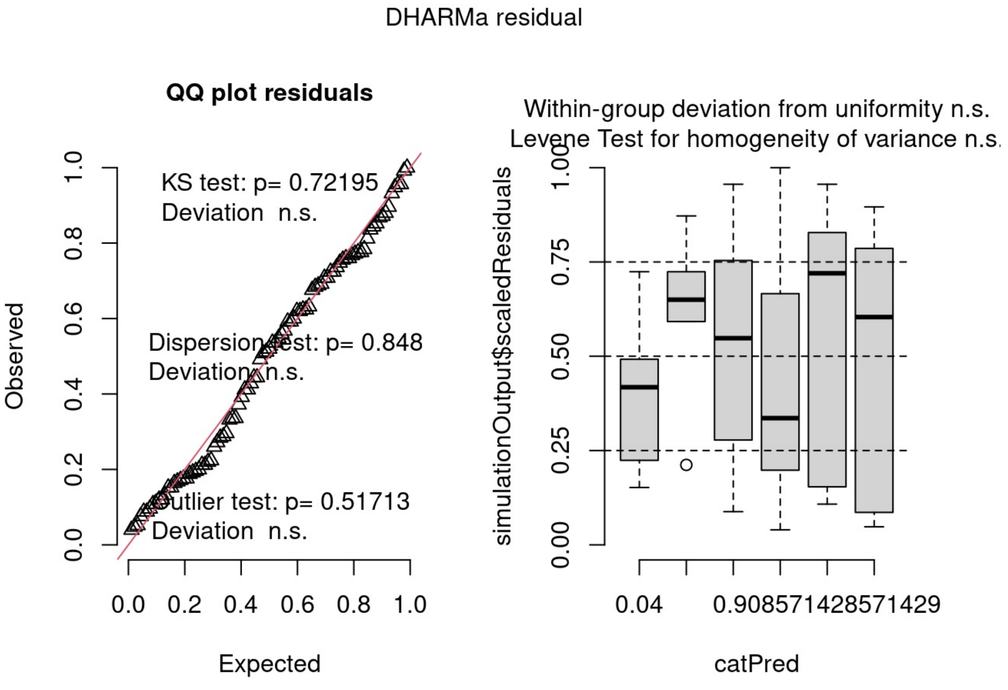

=> no outlier(s)

Estimated marginal means

by group (w FDR correction of pvals)

group	emmean	SE	df	lower.CL	upper.CL
co	0.860	0.042	42.28	0.775	0.945
p	0.797	0.021	42.96	0.754	0.839
mts	0.718	0.049	42.28	0.620	0.817

contrast	estimate	SE	df	t.ratio	p.value	lowerCL	upperCL
co - p	0.063	0.047	42.41	1.343	0.186	-0.054	0.181
co - mts	0.142	0.064	42.28	2.198	0.100	-0.019	0.302
p - mts	0.078	0.053	42.39	1.472	0.186	-0.054	0.211

by surgery

surgery	emmean	SE	df	lower.CL	upper.CL
pre	0.770	0.028	79.00	0.714	0.826
post	0.813	0.028	79.13	0.757	0.869

contrast	estimate	SE	df	t.ratio	p.value	lowerCL	upperCL
pre - post	-0.043	0.034	44.71	-1.282	0.206	-0.111	0.025

Marginal means plot

group

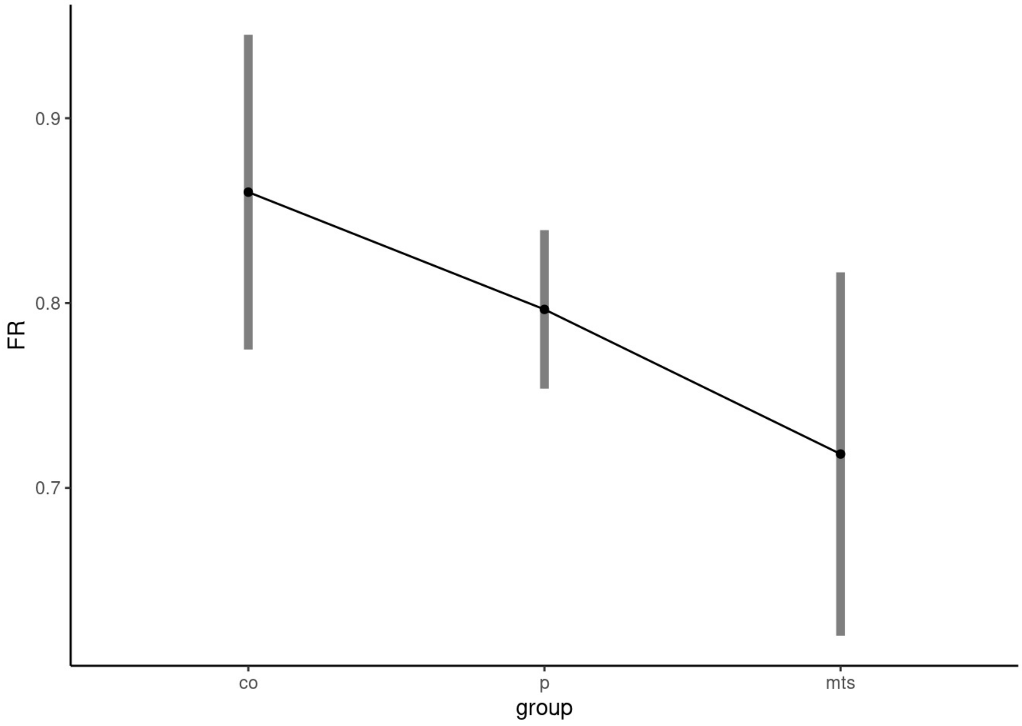

surgery

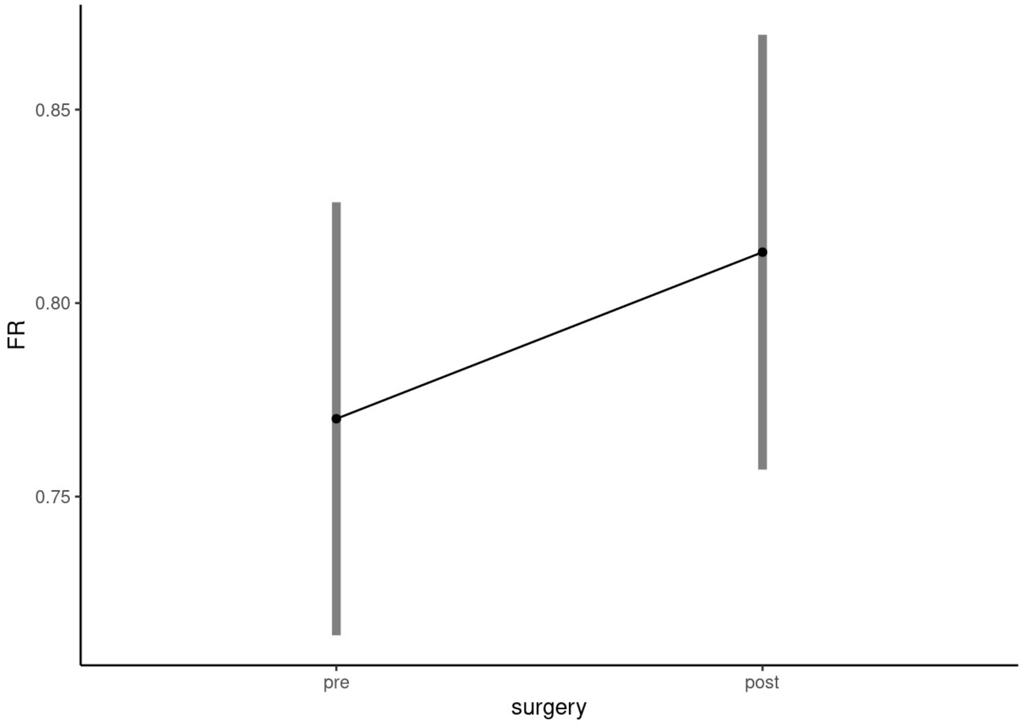

Empirical ROC

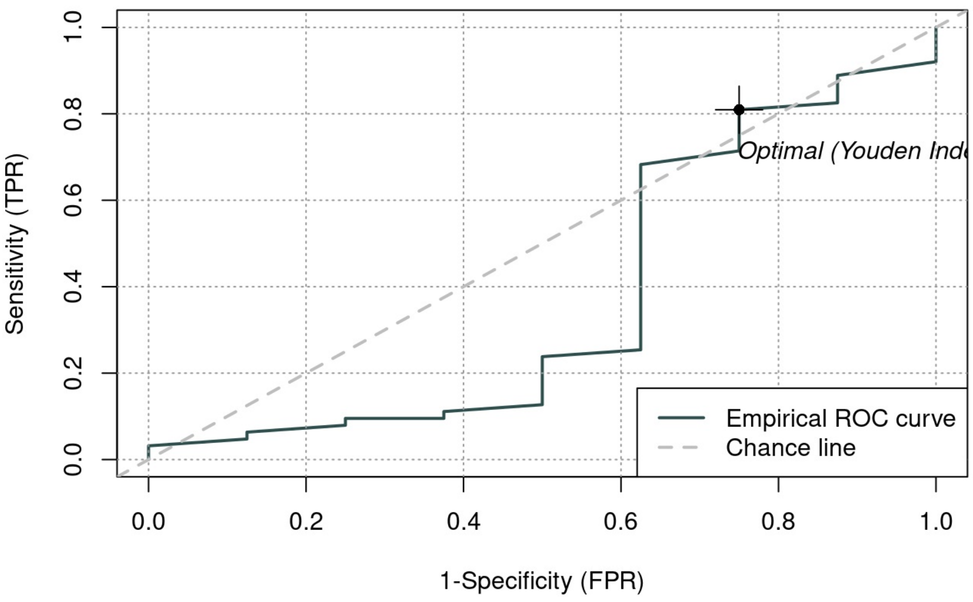


```
png
  2
```

Youden index and cutoff

```
  value     FPR     TPR  cutoff
0.05952 0.75000 0.80952 0.66000
```

95% ci for empirical ROC

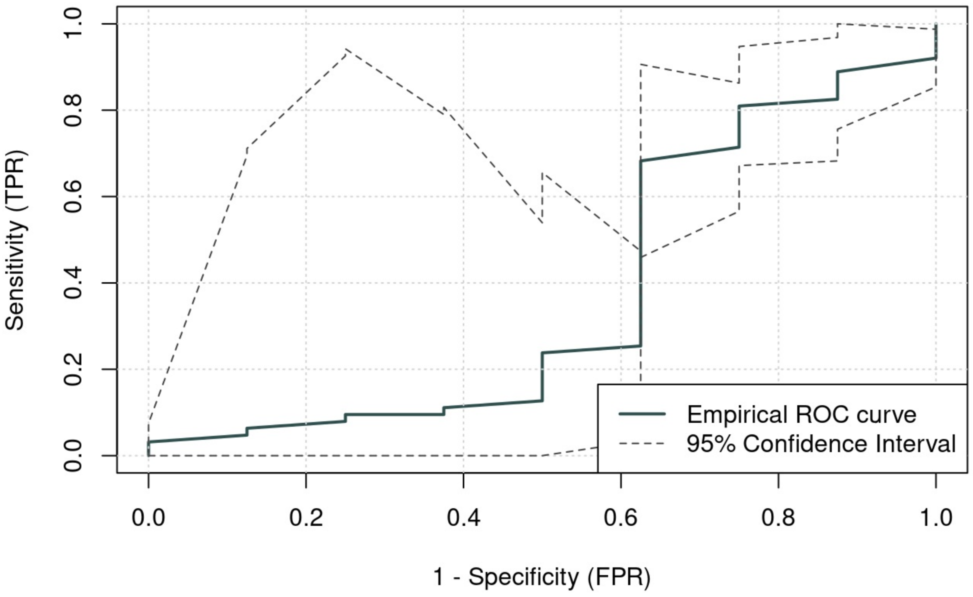


```
png
  2
```

Session info

```
─ Session info ───────────────────────────────────────────────────────────────
 setting  value
 version  R version 4.0.5 (2021-03-31)
 os       CentOS Stream 8
 system   x86_64, linux-gnu
 ui       X11
 language (EN)
 collate  en_US.UTF-8
 ctype    en_US.UTF-8
 tz       Europe/Bratislava
 date     2023-10-09
 pandoc   3.1.1 @ /usr/lib/rstudio/resources/app/bin/quarto/bin/tools/ (via rmarkdown)

─ Packages ───────────────────────────────────────────────────────────────────
 package    * version   date (UTC) lib source
 DescTools  * 0.99.41   2021-04-10 [1] CRAN (R 4.0.5)
 DHARMa     * 0.4.6     2022-09-08 [1] CRAN (R 4.0.5)
 dplyr      * 1.1.2     2023-04-20 [1] CRAN (R 4.0.5)
 emmeans    * 1.7.5     2022-06-22 [1] CRAN (R 4.0.5)
 ggplot2    * 3.4.2     2023-04-03 [1] CRAN (R 4.0.5)
 ggpubr     * 0.4.0.999 2021-07-17 [1] Github (kassambara/ggpubr@ac5a01f)
 ggsci      * 3.0.0     2023-03-08 [1] CRAN (R 4.0.5)
 kableExtra * 1.3.4     2021-02-20 [1] CRAN (R 4.0.5)
 lme4       * 1.1-34    2023-07-04 [1] CRAN (R 4.0.5)
 Matrix     * 1.6-0     2023-07-08 [1] CRAN (R 4.0.5)
 ROCit      * 2.1.1     2020-06-14 [1] CRAN (R 4.0.5)
 sjPlot     * 2.8.12    2022-12-13 [1] Github (strengejacke/sjPlot@4058580)

 [1] /home/mg/R/x86_64-pc-linux-gnu-library/4.0
 [2] /opt/R/4.0.5/lib/R/library

──────────────────────────────────────────────────────────────────────────────
```

report_CDKN2B__Oct9_2023.R

mg

2023-10-09

```
#
#
# Oct 9 2023 Mo -- CDKN2B
# Oct 5 2023 Th -- triplot; spaghetti; lin mixed model - dg, outlier,
#                  emmeans, custom contrasts
# Oct 4 2023 We -- CDK4 ddPCR CNV
#
#
# Eva Lukacova
#
```

CDKN2B

EDA

Triplot (for FR by surgery and group)

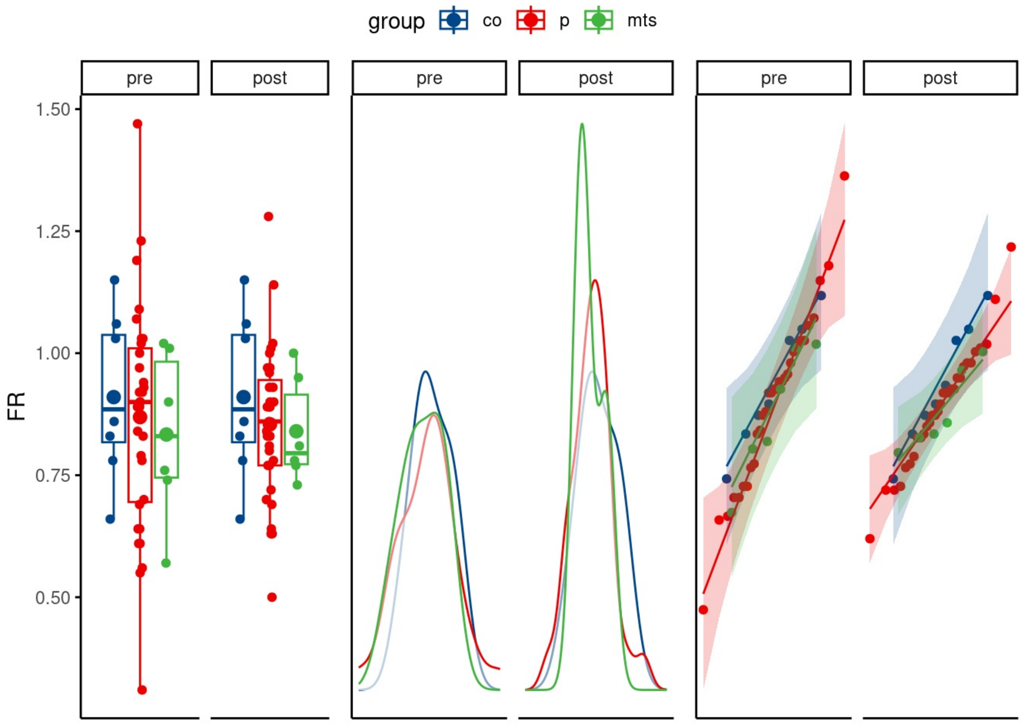

Spaghetti plot for surgery (w group wo co)

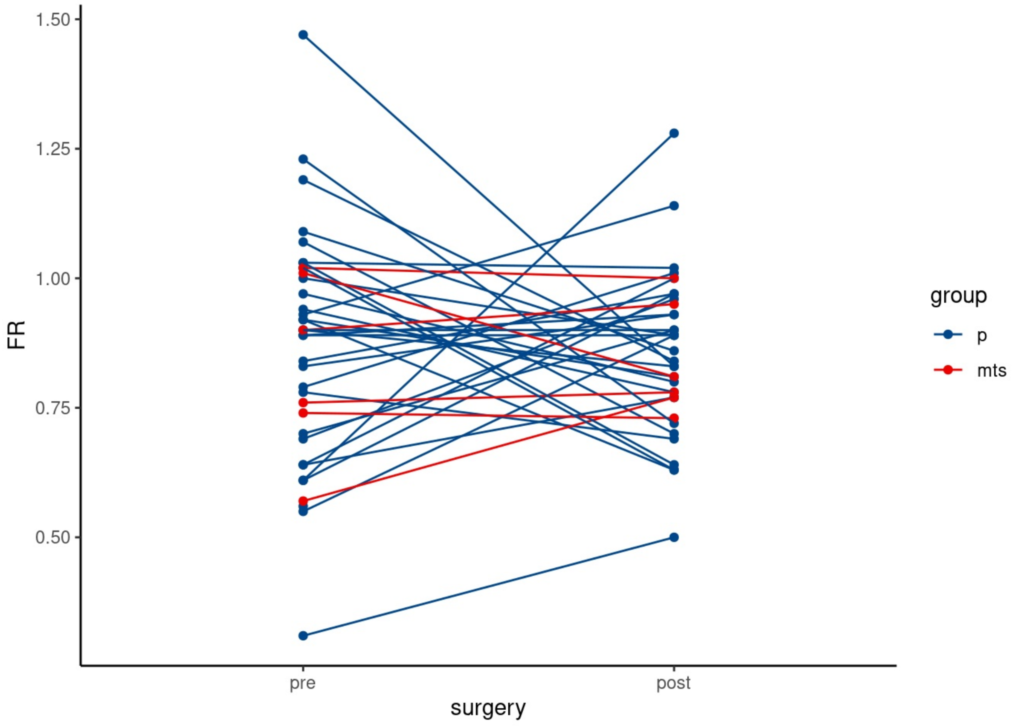

Linear mixed model (to fit Repeated measures ANOVA)

Model fit

	FR		
Predictors	*Estimates*	*CI*	*p*
(Intercept)	0.91	0.81 – 1.02	**<0.001**
group [p]	-0.05	-0.16 – 0.06	0.373
group [mts]	-0.07	-0.22 – 0.07	0.328
surgery [post]	-0.01	-0.08 – 0.06	0.792
**Random Effects**			
σ^2	0.03		
$\tau_{00\ id}$	0.00		
ICC	0.10		
N_{id}	46		
Observations	90		
Marginal R^2 / Conditional R^2	0.014 / 0.115		

Diagnostics

DHARMa residual

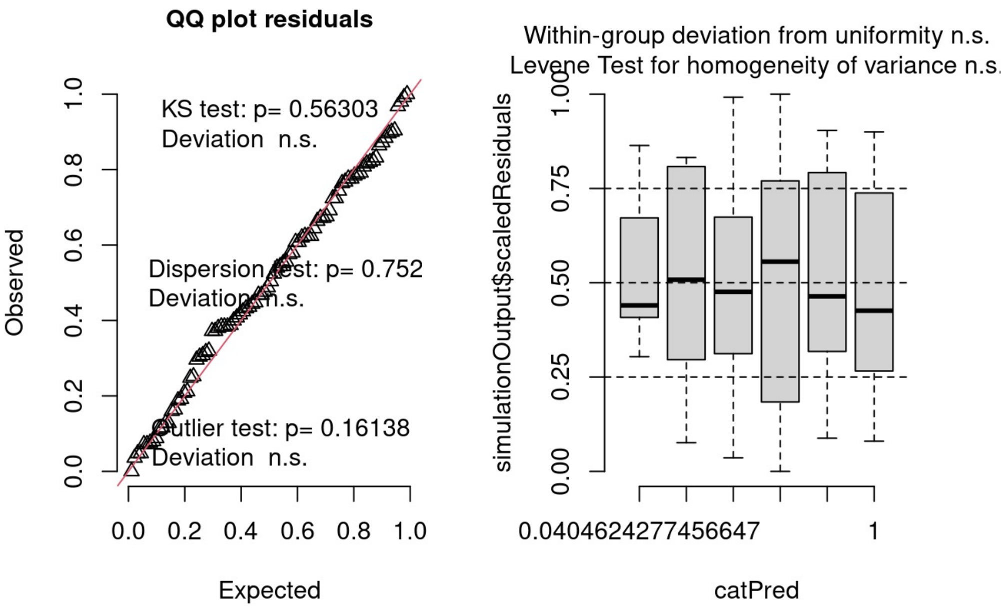

=> no outlier(s)

Estimated marginal means

by group (w FDR correction of pvals)

group	emmean	SE	df	lower.CL	upper.CL
co	0.910	0.049	41.61	0.811	1.009
p	0.861	0.025	42.97	0.811	0.911
mts	0.837	0.056	41.61	0.723	0.950

contrast	estimate	SE	df	t.ratio	p.value	lowerCL	upperCL
co - p	0.049	0.055	41.88	0.896	0.563	-0.087	0.186
co - mts	0.073	0.075	41.61	0.983	0.563	-0.113	0.259
p - mts	0.024	0.062	41.82	0.395	0.695	-0.129	0.178

by surgery

surgery	emmean	SE	df	lower.CL	upper.CL
pre	0.874	0.032	76.17	0.81	0.938
post	0.864	0.032	76.17	0.80	0.928

contrast	estimate	SE	df	t.ratio	p.value	lowerCL	upperCL
pre - post	0.01	0.037	44.37	0.264	0.793	-0.065	0.085

Marginal means plot

group

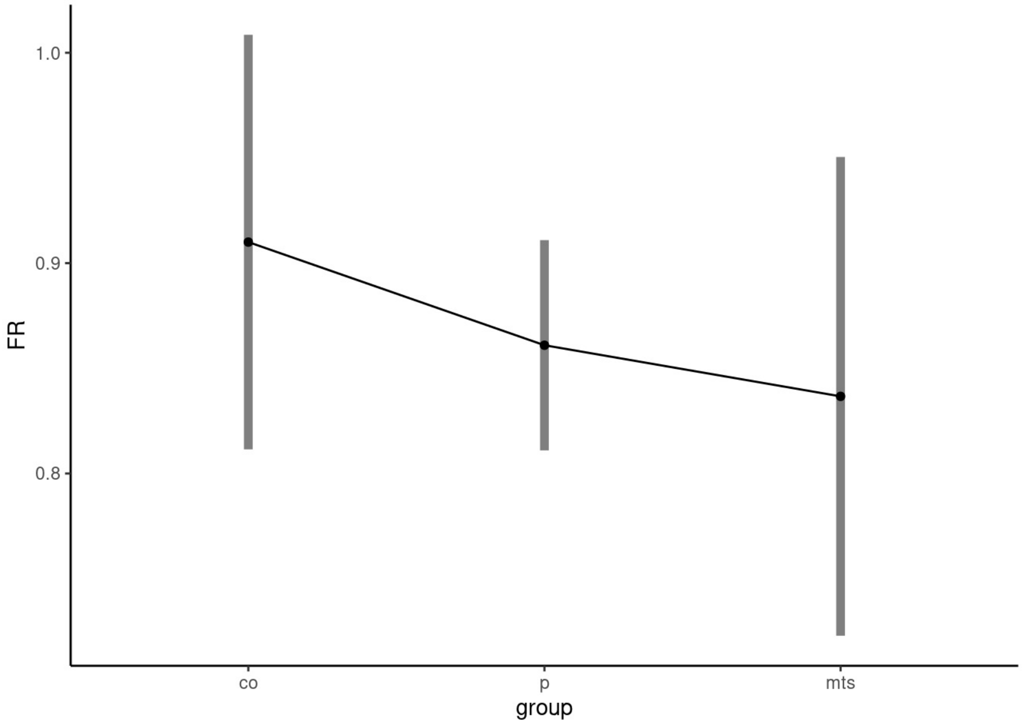

surgery

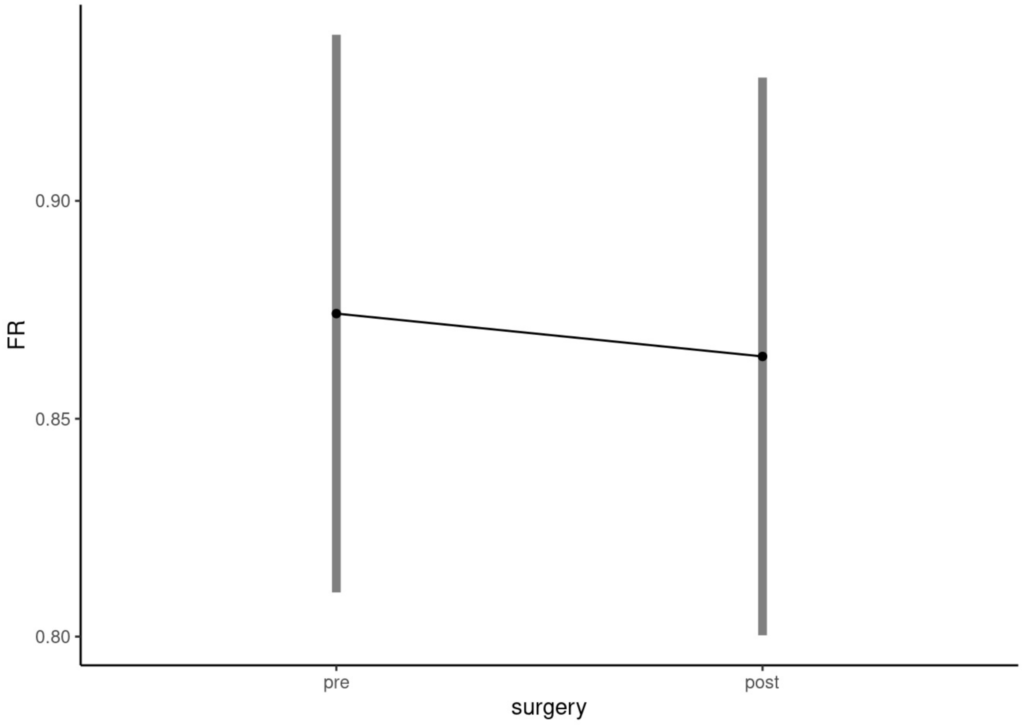

Empirical ROC

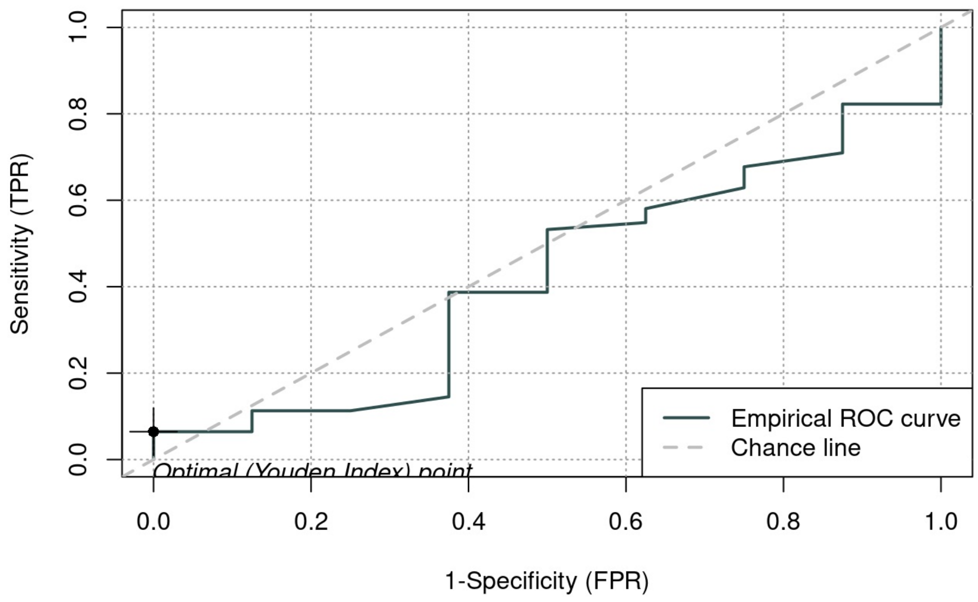


```
png
  2
```

Youden index and cutoff

```
  value     FPR     TPR  cutoff
0.06452 0.00000 0.06452 1.19000
```

95% ci for empirical ROC

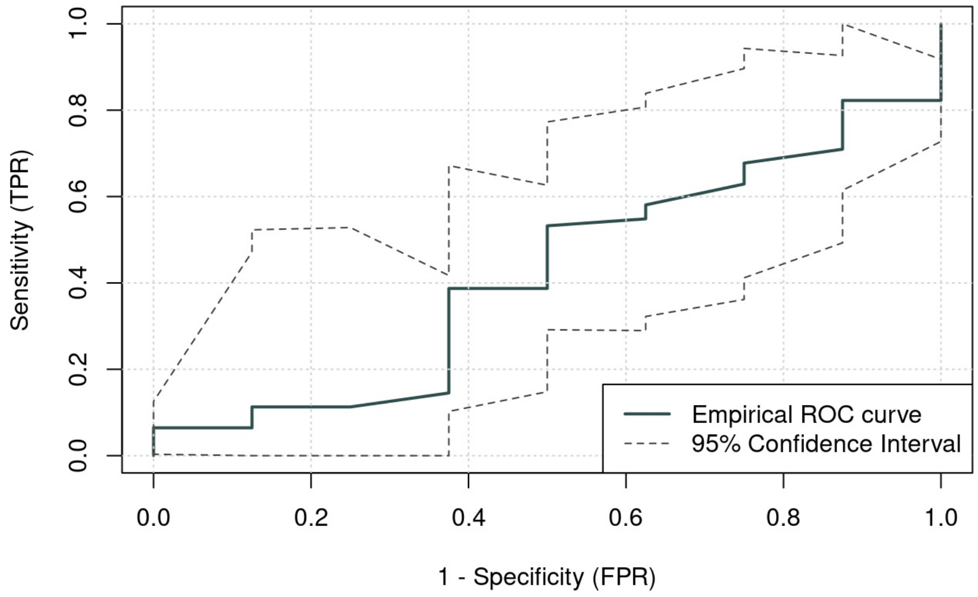


```
png
  2
```

Session info

```
─ Session info ─────────────────────────────────────────────────────────────
 setting  value
 version  R version 4.0.5 (2021-03-31)
 os       CentOS Stream 8
 system   x86_64, linux-gnu
 ui       X11
 language (EN)
 collate  en_US.UTF-8
 ctype    en_US.UTF-8
 tz       Europe/Bratislava
 date     2023-10-09
 pandoc   3.1.1 @ /usr/lib/rstudio/resources/app/bin/quarto/bin/tools/ (via rmarkdown)

─ Packages ─────────────────────────────────────────────────────────────────
 package    * version   date (UTC) lib source
 DescTools  * 0.99.41   2021-04-10 [1] CRAN (R 4.0.5)
 DHARMa     * 0.4.6     2022-09-08 [1] CRAN (R 4.0.5)
 dplyr      * 1.1.2     2023-04-20 [1] CRAN (R 4.0.5)
 emmeans    * 1.7.5     2022-06-22 [1] CRAN (R 4.0.5)
 ggplot2    * 3.4.2     2023-04-03 [1] CRAN (R 4.0.5)
 ggpubr     * 0.4.0.999 2021-07-17 [1] Github (kassambara/ggpubr@ac5a01f)
 ggsci      * 3.0.0     2023-03-08 [1] CRAN (R 4.0.5)
 kableExtra * 1.3.4     2021-02-20 [1] CRAN (R 4.0.5)
 lme4       * 1.1-34    2023-07-04 [1] CRAN (R 4.0.5)
 Matrix     * 1.6-0     2023-07-08 [1] CRAN (R 4.0.5)
 ROCit      * 2.1.1     2020-06-14 [1] CRAN (R 4.0.5)
 sjPlot     * 2.8.12    2022-12-13 [1] Github (strengejacke/sjPlot@4058580)

 [1] /home/mg/R/x86_64-pc-linux-gnu-library/4.0
 [2] /opt/R/4.0.5/lib/R/library

────────────────────────────────────────────────────────────────────────────
```

report_CDK4__Oct9_2023.R

mg

2023-10-09

```
#
#
# Oct 9 2023 Mo -- saving figs; txt
# Oct 5 2023 Th -- triplot; spaghetti; lin mixed model - dg, outlier,
#                  emmeans, custom contrasts
# Oct 4 2023 We -- CDK4 ddPCR CNV
#
#
# Eva Lukacova
#
```

CDK4

EDA

Triplot (for FR by surgery and group)

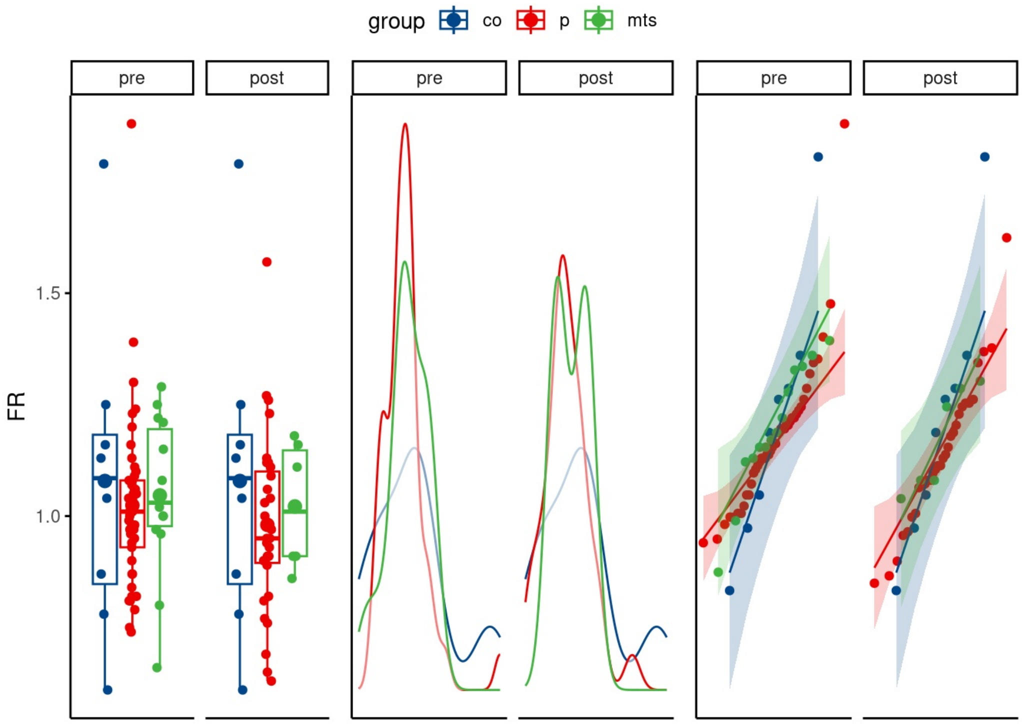

Spaghetti plot for surgery (w group wo co)

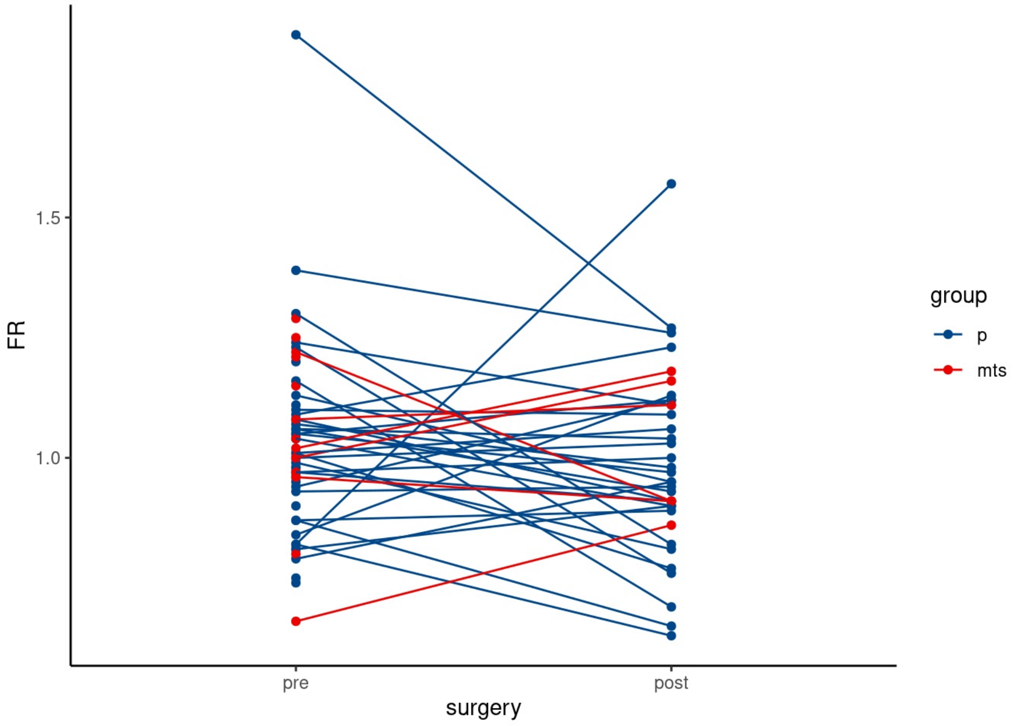

Linear mixed model (to fit Repeated measures ANOVA)

Model fit

	FR		
Predictors	*Estimates*	*CI*	*p*
(Intercept)	1.10	0.96 – 1.23	**<0.001**
group [p]	-0.08	-0.22 – 0.06	0.266
group [mts]	-0.04	-0.21 – 0.13	0.631
surgery [post]	-0.04	-0.10 – 0.02	0.214
**Random Effects**			
σ^2	0.02		
$\tau_{00\ id}$	0.02		
ICC	0.49		
N_{id}	67		
Observations	112		
Marginal R^2 / Conditional R^2	0.025 / 0.506		

Diagnostics

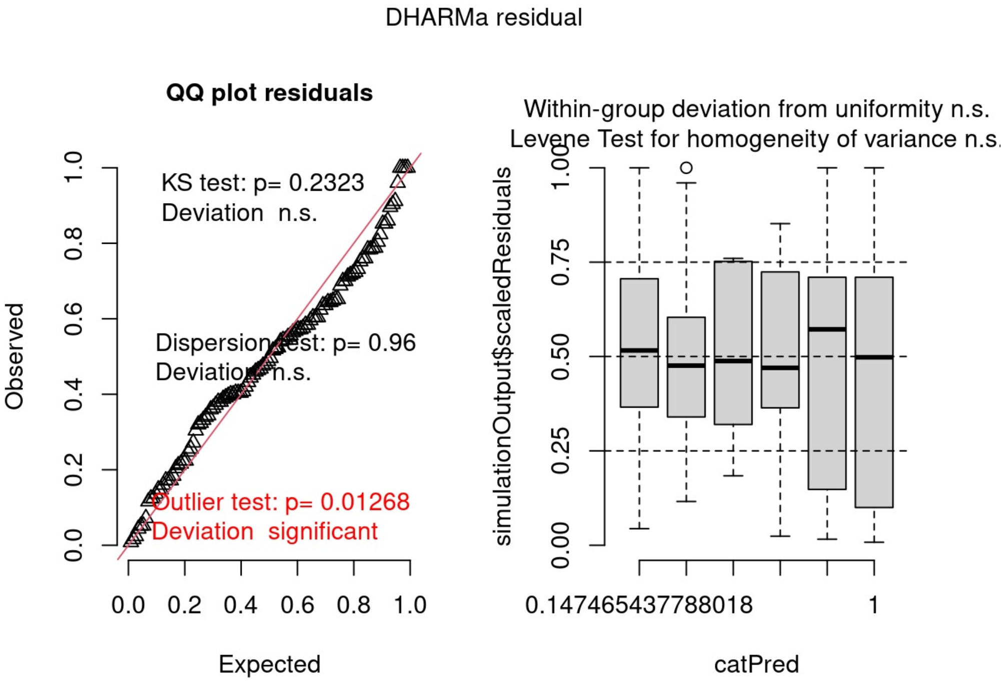

=> outlier(s)

one in co, one in p => search for them

p -> M7

co -> H5

=> remove them

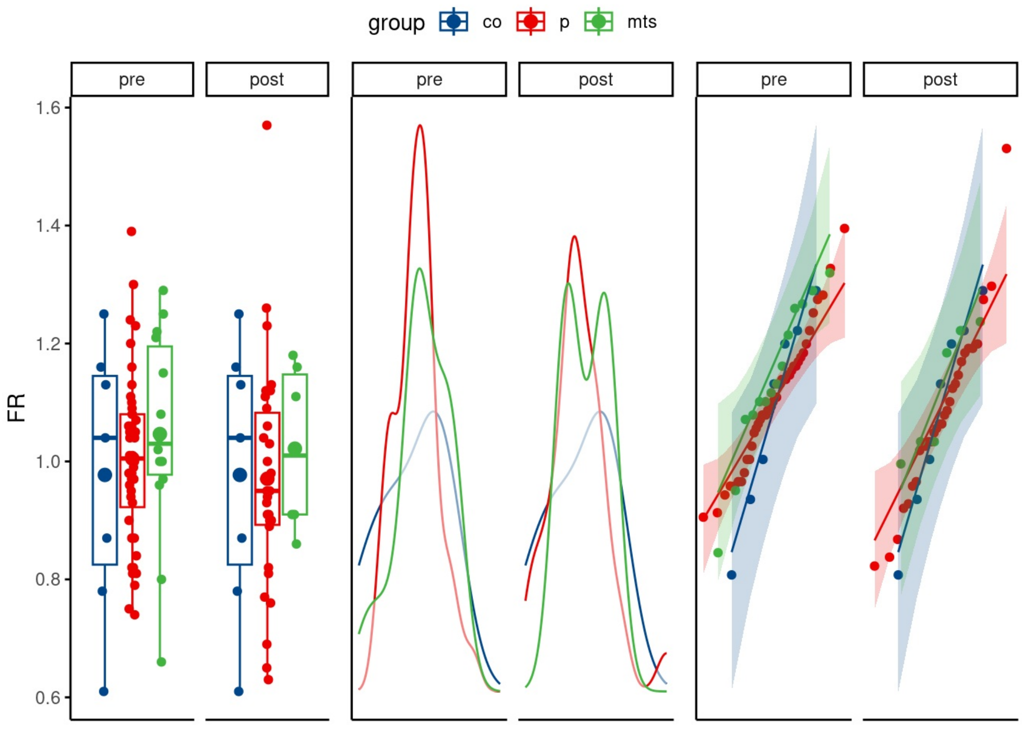

=> rerun wo outliers

Model fit

	FR		
Predictors	*Estimates*	*CI*	*p*
(Intercept)	0.99	0.88 – 1.10	**<0.001**
group [p]	0.01	-0.10 – 0.12	0.874
group [mts]	0.06	-0.07 – 0.19	0.374
surgery [post]	-0.03	-0.09 – 0.03	0.333

Random Effects

σ^2	0.02
$\tau_{00\ id}$	0.01
ICC	0.29
N_{id}	65
Observations	108
Marginal R^2 / Conditional R^2	0.023 / 0.309

Diagnostics

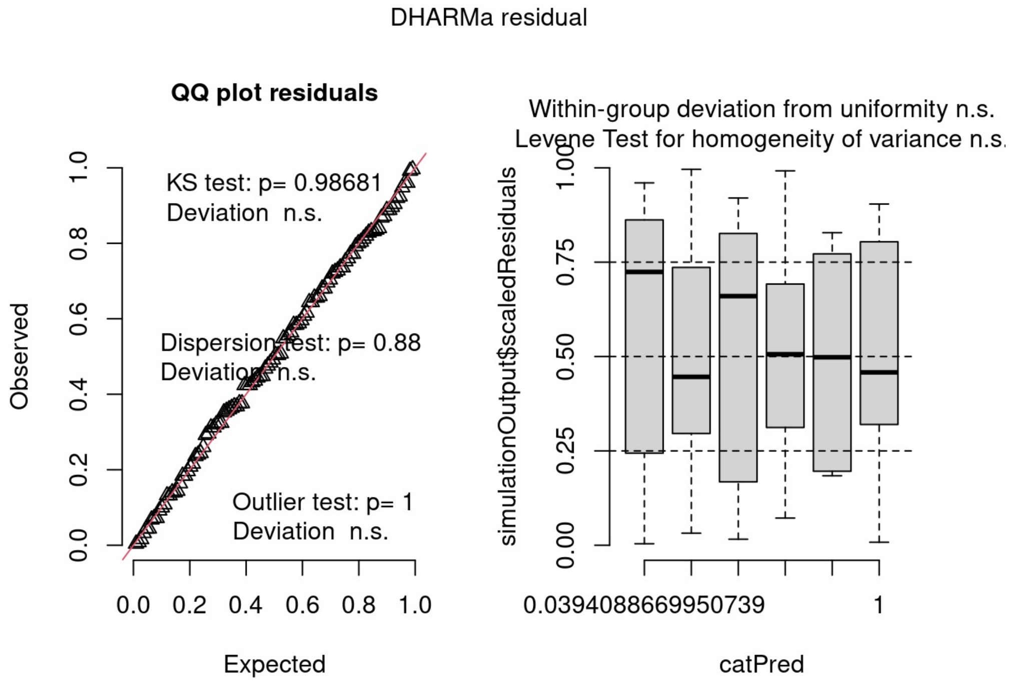

Estimated marginal means

by group (w FDR correction of pvals)

group	emmean	SE	df	lower.CL	upper.CL
co	0.977	0.053	48.88	0.871	1.083
p	0.986	0.023	59.52	0.941	1.032
mts	1.038	0.042	71.40	0.953	1.122

contrast	estimate	SE	df	t.ratio	p.value	lowerCL	upperCL
co - p	-0.009	0.057	50.40	-0.159	0.874	-0.151	0.133
co - mts	-0.060	0.068	56.85	-0.892	0.564	-0.227	0.107
p - mts	-0.051	0.048	67.74	-1.077	0.564	-0.168	0.066

by surgery

surgery	emmean	SE	df	lower.CL	upper.CL
pre	1.015	0.026	82.95	0.963	1.067
post	0.986	0.030	96.69	0.926	1.045

contrast	estimate	SE	df	t.ratio	p.value	lowerCL	upperCL
pre - post	0.029	0.03	53.06	0.968	0.337	-0.031	0.089

Marginal means plot

group

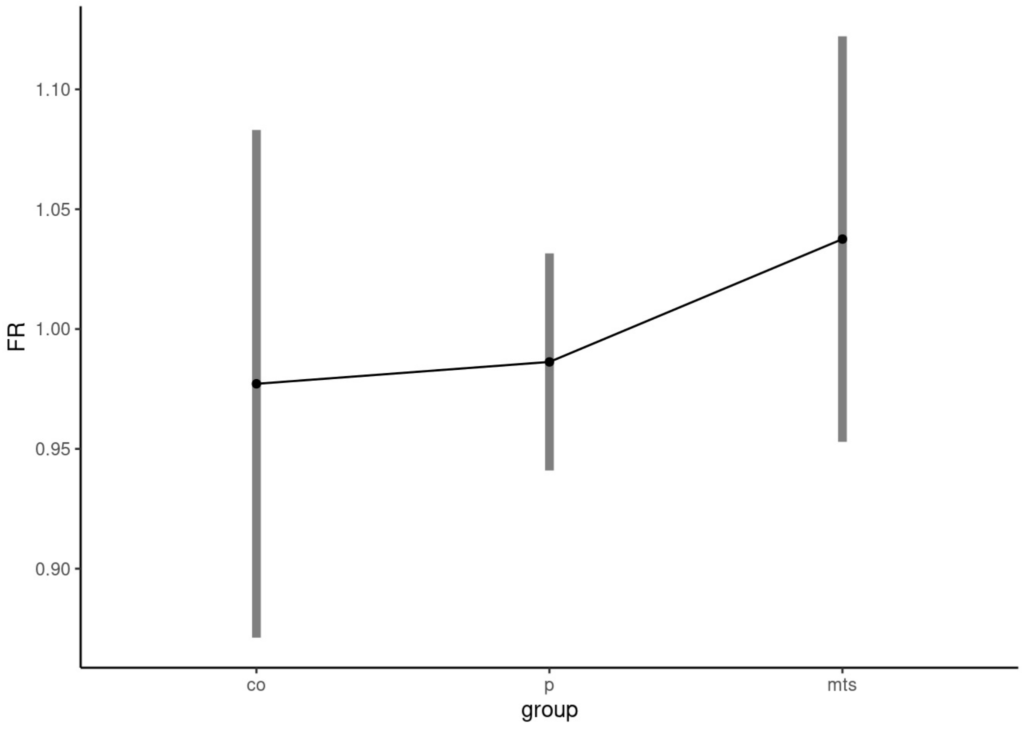

surgery

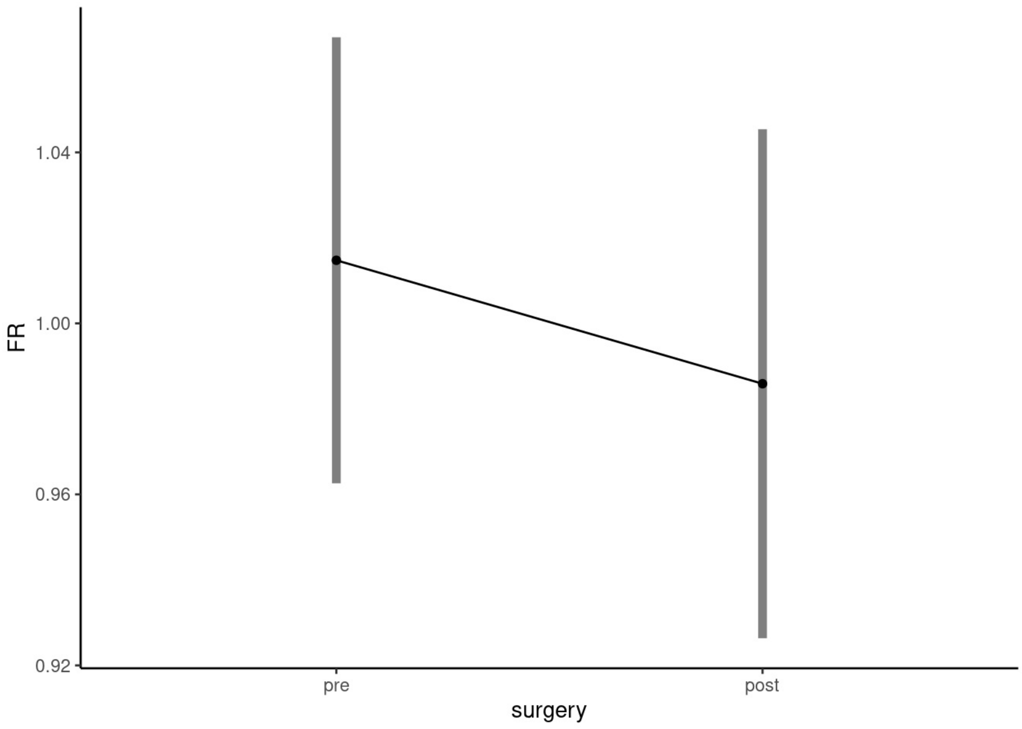

Empirical ROC

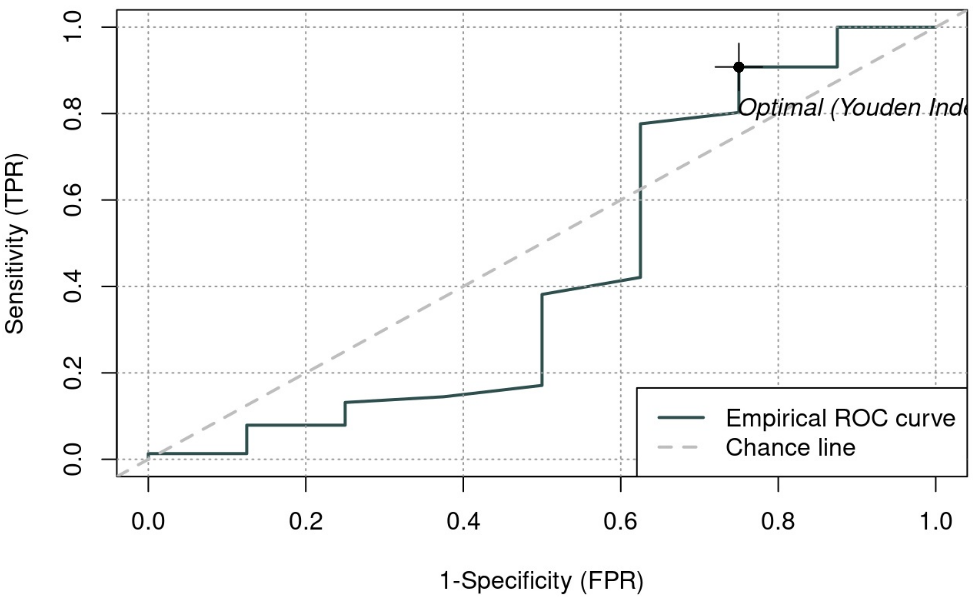


```
png 
  2 
```

Youden index and cutoff

```
 value    FPR    TPR cutoff 
0.1579 0.7500 0.9079 0.7900 
```

95% ci for empirical ROC

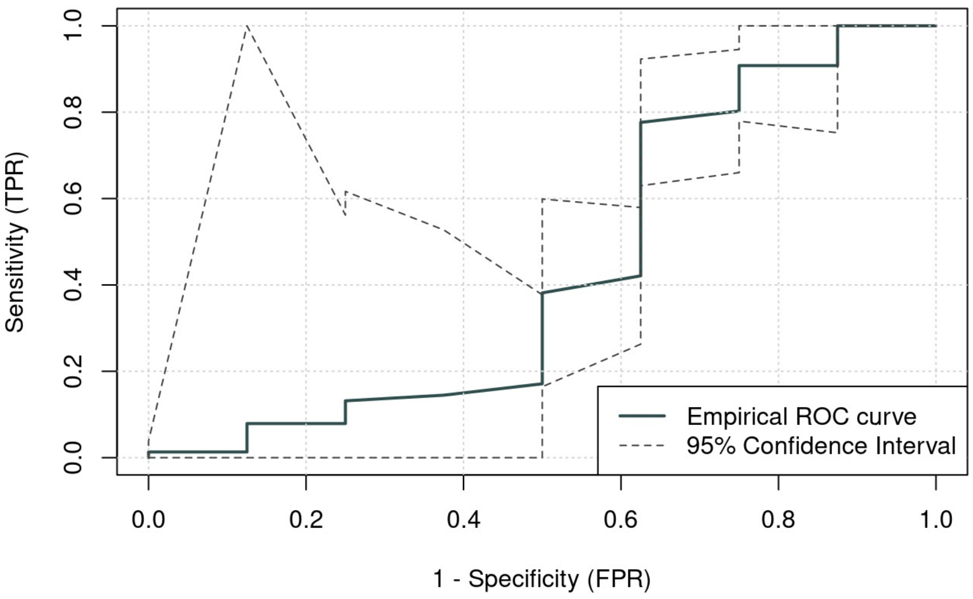


```
png 
  2 
```

Data analysis

Data were explored and analyzed in R [1], ver. 4.0.5, with the aid of libraries listed below at Session info.

Distribution of the Final Ratio (FR) was visualized by boxplot overlaid with swarmplot, density plot and Quantile-Quantile plot with the 95% confidence band constructed by bootstrap. Change of FR between pre- and post-surgery stages was visualized by the spaghetti plot.

Linear mixed model of the form Fr ~ group + surgery + (1|id) was fitted to the data by REML method. There, group was a factor with three levels: control, primary, metastasis and surgery was a factor with two levels: pre, post. The fitted model was subjected to diagnostic analysis of residuals. If outliers were detected (two outlier for CDK4, none for CDKN2A and CDKN2B) then they were excluded from considerations and the model was refit and subjected to diagnostics. Effect size was quantified by the Adjusted R^2.

The fitted linear mixed model was used to estimate the marginal means. Post-hoc pairwise comparisons were performed with the Benjamini-Hochbergh correction of p-values in the case of the comparisons for group factor. The marginal means together with their 95% confidence interval were visualized by the marginal means plot.

[1] R Core Team (2021). R: A language and environment for statistical computing. R Foundation for Statistical Computing, Vienna, Austria. URL https://www.R-project.org/ (https://www.R-project.org/).

Session info

```
─ Session info ───────────────────────────────────────────────────────────────
 setting  value
 version  R version 4.0.5 (2021-03-31)
 os       CentOS Stream 8
 system   x86_64, linux-gnu
 ui       X11
 language (EN)
 collate  en_US.UTF-8
 ctype    en_US.UTF-8
 tz       Europe/Bratislava
 date     2023-10-09
 pandoc   3.1.1 @ /usr/lib/rstudio/resources/app/bin/quarto/bin/tools/ (via rmarkdown)

─ Packages ───────────────────────────────────────────────────────────────────
 package    * version   date (UTC) lib source
 DescTools  * 0.99.41   2021-04-10 [1] CRAN (R 4.0.5)
 DHARMa     * 0.4.6     2022-09-08 [1] CRAN (R 4.0.5)
 dplyr      * 1.1.2     2023-04-20 [1] CRAN (R 4.0.5)
 emmeans    * 1.7.5     2022-06-22 [1] CRAN (R 4.0.5)
 ggplot2    * 3.4.2     2023-04-03 [1] CRAN (R 4.0.5)
 ggpubr     * 0.4.0.999 2021-07-17 [1] Github (kassambara/ggpubr@ac5a01f)
 ggsci      * 3.0.0     2023-03-08 [1] CRAN (R 4.0.5)
 kableExtra * 1.3.4     2021-02-20 [1] CRAN (R 4.0.5)
 lme4       * 1.1-34    2023-07-04 [1] CRAN (R 4.0.5)
 Matrix     * 1.6-0     2023-07-08 [1] CRAN (R 4.0.5)
 ROCit      * 2.1.1     2020-06-14 [1] CRAN (R 4.0.5)
 sjPlot     * 2.8.12    2022-12-13 [1] Github (strengejacke/sjPlot@4058580)

 [1] /home/mg/R/x86_64-pc-linux-gnu-library/4.0
 [2] /opt/R/4.0.5/lib/R/library

──────────────────────────────────────────────────────────────────────────────
```
