# Supplementary material for: Novel liquid biopsy CNV biomarkers in malignant melanoma
Source: Sci Rep. 2024 Jul 9;14:15786. doi: 10.1038/s41598-024-65928-y (PMC11233564; doi:10.1038/s41598-024-65928-y)
Supplement: Supplementary file 3 — Supplementary Tables. [file 41598_2024_65928_MOESM3_ESM.docx]

Supplementary tables

| Patient number | Gender | Age | Type MM | Located at | Clark | Tissue | Pre-surgery  plasma | Post-surgery plasma | Number of days* | BRAF tissue | BRAF plasma |
| --- | --- | --- | --- | --- | --- | --- | --- | --- | --- | --- | --- |
| **1-MM** | M | 60 | SSM | C43.4 | IV | ✓ | ✓ | ✓ | 78 | WT |  |
| **2-MM** | F | 76 | MM-NS | C43.5 | IV | ✓ | ✓ | ✓ | 68 | WT | BRAFV600E |
| **3-MM** | M | 64 | SSM | C43.5 | IV | ✓ | ✓ | ✓ | 19 | WT | BRAFV600E |
| **4-MM** | F | 74 | ALM | C43.6 | II | ✓ | ✓ | ✓ | 104 | WT |  |
| **5-MM** | F | 74 | NM | C43.6 | IV | ✓ | ✓ | ✓ | 21 | WT |  |
| **6-MM** | F | 68 | MM-NS | C44.6 | III | ✓ | ✓ | ✓ | 19 | WT |  |
| **7-MM** | F | 72 | SSM | C43.6 | III | ✓ | ✓ | ✓ | 36 | WT |  |
| **8-MM** | F | 40 | SSM | C43.5 | III | ✓ | ✓ | ✓ | 141 | BRAFV600E | BRAFV600E |
| **9-MM** | F | 69 | SSM | C43.6 | V | ✓ | ✓ | ✓ | 118 | BRAFV600E | BRAFV600E |
| **10-MM** | F | 58 | SSM | C43.7 | IV | ✓ | ✓ | ✓ | 119 | BRAFV600E | BRAFV600E |
| **11-MM** | F | 29 | SSM | C43.7 | IV | ✓ | ✓ | ✓ | 192 | WT |  |
| **12-MM** | M | 65 | SSM | C43.4 | III | ✓ | ✓ | ✓ | 135 | WT |  |
| **13-MM** | M | 58 | MM-NS | C43.5 | IV | ✓ | ✓ | ✓ | 267 | WT |  |
| **14-MM** | M | 79 | LMM | C43.4 | IV | ✓ | ✓ | ✓ | 103 | WT | BRAFV600E |
| **15-MM** | F | 81 | MM-NS | C43.5 | II | ✓ | ✓ | ✓ | 53 | WT | BRAFV600E |
| **16-MM** | M | 70 | SSM | C43.6 | IV | ✓ | ✓ | ✓ | 92 | WT | BRAFV600E |
| **17-MM** | M | 78 | SSM | C43.5 | IV | ✓ | ✓ | ✓ | 110 | BRAFV600E | BRAFV600E |
| **18-MM** | F | 74 | ALM | C43.6 | IV | ✓ | ✓ | ✓ | 19 | WT |  |
| **19-MM** | M | 62 | SSM | C43.5 | IV | ✓ | ✓ | ✓ | 379 | BRAFV600E | BRAFV600E |
| **20-MM** | M | 83 | SSM | C43.5 | IV | ✓ | ✓ | ✓ | 85 | BRAFV600E |  |
| **21-MM** | F | 75 | SSM | C43.6 | II | ✓ | ✓ | ✓ | 108 | WT | BRAFV600E |
| **22-MM** | M | 68 | ALM | C43.7 | V | ✓ | ✓ | ✓ | 106 | WT | BRAFV600E |
| **23-MM** | M | 62 | MM-NS | C43.5 | III | ✓ | ✓ | ✓ | 99 | WT |  |
| **24-MM** | F | 80 | ALM | C43.7 | V | ✓ | ✓ | ✓ | 146 | WT | BRAFV600E |
| **25-MM** | M | 69 | MM-NS | C43.5 | II | ✓ | ✓ | ✓ | 106 | WT | BRAFV600E |
| **26-MM** | M | 54 | MM-NS | C43.7 | IV | ✓ | ✓ | ✓ | 97 | WT |  |
| **27-MM** | M | 55 | SSM | C43.3 | III | ✓ | ✓ | ✓ | 33 | WT | BRAFV600E |
| **28-MM** | M | 43 | SSM | C43.5 | IV | ✓ | ✓ | ✓ | 167 | BRAFV600E | BRAFV600E |
| **29-MM** | M | 64 | SSM | C43.5 | IV | ✓ | ✓ | ✓ | 15 | WT |  |
| **30-MM** | M | 76 | MM-NS | C43.6 | IV | ✓ | ✓ | ✓ | 124 | N/A |  |
| **31-MM** | F | 64 | SSM | C43.6 | IV | ✓ | ✓ | ✓ | 131 | WT | BRAFV600E |
| **32-MM** | F | 70 | SSM | C43.6 | IV | ✓ | ✓ | ✓ | 92 | WT |  |
| **33-MM** | M | 56 | MM-NS | C43.6 | IV | ✓ | ✓ | N/A | N/A | WT |  |
| **34-MM** | F | 82 | SSM | C43.6 | IV | ✓ | ✓ | N/A | N/A | WT |  |
| **35-MM** | F | 89 | ALM | C43.7 | V | ✓ | ✓ | N/A | N/A | WT |  |
| **36-MM** | M | 47 | SSM | C43.5 | II | ✓ | ✓ | N/A | N/A | WT |  |
| **37-MM** | F | 84 | ALM | C43.7 | V | ✓ | ✓ | N/A | N/A | WT |  |
| **38-MM** | M | 85 | SSM | C43.3 | II | ✓ | ✓ | N/A | N/A | BRAFV600E |  |
| **39-MM** | M | 58 | MM-NS | C43.7 | IV | ✓ | ✓ | N/A | N/A | WT |  |
| **40-MM** | M | 61 | NM | C43.5 | IV | ✓ | ✓ | N/A | N/A | BRAFV600E | BRAFV600E |
| **41-MM** | M | 68 | MM-NS | C43.5 | III | ✓ | ✓ | N/A | N/A | BRAFV600E | BRAFV600E |
| **42-MM** | F | 47 | MM-NS | C43.5 | IV | ✓ | ✓ | N/A | N/A | BRAFV600E | BRAFV600E |
| **43-MM** | M | 51 | SSM | C43.5 | IV | ✓ | ✓ | N/A | N/A | BRAFV600E | BRAFV600E |
| **44-MM** | M | 56 | NM | C43.5 | IV | ✓ | ✓ | N/A | N/A | N/A |  |
| **45-MM** | M | 72 | SSM | C43.6 | IV | ✓ | ✓ | N/A | N/A | N/A |  |
| **46-MM** | M | 83 | LMM | C43.5 | IV | ✓ | ✓ | N/A | N/A | WT |  |

**Supplementary Table 1: Primary MM samples.** *Number of days between surgery and collection after surgery - BRAF status was detected by ddPCR and published: Malicherova *et al.,* 2018; Burjanivova *et al.,* 2019.

| Patient number | Gender | Age | Type MTS | Pre-surgery plasma | Post-surgery  plasma | Number of days* | BRAF plasma |
| --- | --- | --- | --- | --- | --- | --- | --- |
| **47-MM** | M | 71 | EN | ✓ | ✓ | 104 |  |
| **48MM** | F | 75 | EN | ✓ | ✓ | 79 |  |
| **49MM** | M | 76 | LU | ✓ | ✓ | 96 |  |
| **50MM** | F | 76 | Cutaneous MTS | ✓ | ✓ | 35 |  |
| **51MM** | F | 77 | LU | ✓ | ✓ | 19 | BRAFV600E |
| **52MM** | M | 62 | LU | ✓ | ✓ | 19 |  |
| **53MM** | F | 72 | Skin MTS | ✓ | N/A | N/A | BRAFV600E |
| **54MM** | F | 57 | Subcutaneous MTS | ✓ | N/A | N/A |  |
| **55MM** | M | 74 | LU | ✓ | N/A | N/A | BRAFV600E |
| **56MM** | M | 78 | LU | ✓ | N/A | N/A |  |
| **57MM** | M | 63 | LU | ✓ | N/A | N/A |  |
| **58MM** | M | 67 | LU | ✓ | N/A | N/A | BRAFV600E |
| **59MM** | M | 60 | LU | ✓ | N/A | N/A |  |
| **60MM** | M | 60 | LU | ✓ | N/A | N/A |  |

**Supplementary Table 2: Metastatic MM samples.** *Number of days between surgery and collection after surgery - BRAF status was detected by ddPCR and published: Malicherova *et al.,* 2018; Burjanivova *et al.,* 2019.

| ***Patient*** | ***Type*** | ***Diagnosis*** | ***Clark*** | ***BRAFV600E*** | ***CDKN2A*** | ***CDKN2B*** | ***CDK4*** |
| --- | --- | --- | --- | --- | --- | --- | --- |
| **1MM** | SSM | C43.4 | IV | WT |  |  |  |
| **2MM** | MM-NS | C43.5 | IV | WT |  |  |  |
| **3MM** | SSM | C43.5 | IV | WT |  |  |  |
| **4MM** | ALM | C43.6 | II | WT |  |  |  |
| **5MM** | NM | C43.6 | IV | WT |  |  |  |
| **6MM** | MM-NS | C44.6 | III | WT |  |  |  |
| **7MM** | SSM | C43.6 | III | WT |  |  |  |
| **8MM** | SSM | C43.5 | III | BRAFV600E |  |  |  |
| **9MM** | SSM | C43.6 | V | BRAFV600E |  |  |  |
| **10MM** | SSM | C43.7 | IV | BRAFV600E |  |  |  |
| **11MM** | SSM | C43.7 | IV | WT |  |  |  |
| **12MM** | SSM | C43.4 | III | WT |  |  |  |
| **13MM** | MM-NS | C43.5 | IV | WT |  |  |  |
| **14MM** | LMM | C43.4 | IV | WT |  |  |  |
| **15MM** | MM-NS | C43.5 | II | WT |  |  |  |
| **16MM** | SSM | C43.6 | IV | WT |  |  |  |
| **17MM** | SSM | C43.5 | IV | BRAFV600E |  |  |  |
| **18MM** | ALM | C43.6 | IV | WT |  |  |  |
| **19MM** | SSM | C43.5 | IV | BRAFV600E |  |  |  |
| **20MM** | SSM | C43.5 | IV | BRAFV600E |  |  |  |
| **21MM** | SSM | C43.6 | II | WT |  |  |  |
| **22MM** | ALM | C43.7 | V | WT |  |  |  |
| **23MM** | MM-NS | C43.5 | III | WT |  |  |  |
| **24MM** | ALM | C43.7 | V | WT |  |  |  |
| **25MM** | MM-NS | C43.5 | II | WT |  |  |  |
| **26MM** | MM-NS | C43.7 | IV | WT |  |  |  |
| **27MM** | SSM | C43.3 | III | WT |  |  |  |
| **28MM** | SSM | C43.5 | IV | BRAFV600E |  |  |  |
| **29MM** | SSM | C43.5 | IV | WT |  |  |  |
| **30MM** | MM-NS | C43.6 | IV | N/A |  |  |  |
| **31MM** | SSM | C43.6 | IV | WT |  |  |  |
| **32MM** | SSM | C43.6 | IV | WT |  |  |  |
| **33MM** | MM-NS | C43.6 | IV | WT |  |  |  |
| **34MM** | SSM | C43.6 | IV | WT |  |  |  |
| **35MM** | ALM | C43.7 | V | WT |  |  |  |
| **36MM** | SSM | C43.5 | II | WT |  |  |  |
| **37MM** | ALM | C43.7 | V | WT |  |  |  |
| **38MM** | SSM | C43.3 | II | BRAFV600E |  |  |  |
| **39MM** | MM-NS | C43.7 | IV | WT |  |  |  |
| **40MM** | NM | C43.5 | IV | BRAFV600E |  |  |  |
| **41MM** | MM-NS | C43.5 | III | BRAFV600E |  |  |  |
| **42MM** | MM-NS | C43.5 | IV | BRAFV600E |  |  |  |
| **43MM** | SSM | C43.5 | IV | BRAFV600E |  |  |  |
| **44MM** | NM | C43.5 | IV | N/A |  |  |  |
| **45MM** | SSM | C43.6 | IV | N/A |  |  |  |
| **46MM** | LMM | C43.5 | IV | WT |  |  |  |

**Supplementary Table 3. MLPA analysis result of FFPE MM of primary tumor tissues (N=46).** Red outline shows deletion, green shows amplification, and gray color indicates no CNV present.

| ***Occurrence***  ***Tumor*** | ***Occurrence Healthy*** | ***Cytoband*** | ***Location*** | ***Length*** |
| --- | --- | --- | --- | --- |
| 7 | 0 | 6q27 | Chr6: 167,996,241 - 168,101,511 | 105270 |
| 5 | 0 | 4p16.1 | Chr4: 9,210,657 - 9,364,721 | 154064 |
| 5 | 0 | 6q27 | Chr6: 167,155,247 - 170,584,692 | 3429445 |
| 5 | 0 | 10p15.3 | Chr10: 274,190 - 689,668 | 415478 |
| 5 | 0 | 10q22.3 | Chr10: 79,347,469 - 79,445,624 | 98155 |
| 5 | 0 | 13q34 | Chr13: 112,894,378 - 113,267,108 | 372730 |
| 5 | 0 | 18q23 | Chr18: 79,069,285 - 79,378,287 | 309002 |
| 5 | 0 | 20q11.21-q13.12 | Chr20: 32,358,330 - 47,786,616 | 15428286 |
| 5 | 0 | 22q13.33 | Chr22: 49,773,283 - 50,783,667 | 1010384 |
| 4 | 0 | 3q12.2 | Chr3: 100,709,295 - 100,748,964 | 39669 |
| 4 | 0 | 5p15.33 | Chr5: 191,495 - 1,887,236 | 1695741 |
| 4 | 0 | 6q25.3-q27 | Chr6: 157,289,025 - 167,139,141 | 9850116 |
| 4 | 0 | 7p22.3 | Chr7: 290,170 - 2,775,500 | 2746483 |
| 4 | 0 | 7q36.1-q36.3 | Chr7: 149,472,696 - 159,144,867 | 9672171 |
| 4 | 0 | 8p21.3 | Chr8: 22,048,995 - 22,232,101 | 183106 |
| 4 | 0 | 8p23.1 | Chr8: 10,482,878 - 12,756,073 | 2273195 |
| 4 | 0 | 8p23.3 | Chr8: 406,428 - 2,165,552 | 1759124 |
| 4 | 0 | 9q31.1 | Chr9: 102,995,311 - 104,777,764 | 1782453 |
| 4 | 0 | 10p15.3 | Chr10: 134,465 - 1,132,384 | 997919 |
| 4 | 0 | 10q22.3 | Chr10: 79,512,533 - 79,615,455 | 102922 |
| 4 | 0 | 10q26.3 | Chr10: 132,186,948 - 133,523,558 | 1336610 |
| 4 | 0 | 12p11.22 | Chr12: 28,133,249 - 28,581,511 | 448262 |
| 4 | 0 | 12q24.33 | Chr12: 131,828,393 - 132,956,306 | 1127913 |
| 4 | 0 | 13q34 | Chr13: 110,712,736 - 114,337,626 | 3624890 |
| 4 | 0 | 14q32.2 | Chr14: 99,500,190 - 100,144,236 | 644046 |
| 4 | 0 | 14q32.33 | Chr14: 104,136,454 - 105,530,202 | 1393748 |
| 4 | 0 | 15q14-q22.31 | Chr15: 39,588,357 - 64,072,033 | 24483676 |
| 4 | 0 | 15q24.1-q25.1 | Chr15: 73,873,564 - 79,472,304 | 5598740 |
| 4 | 0 | 15q26.3 | Chr15: 99,715,697 - 100,342,005 | 626308 |
| 4 | 0 | 16p11.2-p12.1 | Chr16: 27,224,994 - 32,676,165 | 5451171 |
| 4 | 0 | 16p13.3 | Chr16: 58,059 - 3,142,804 | 3084745 |
| 4 | 0 | 16q11.2-q13 | Chr16: 46,656,132 - 57,284,672 | 10628540 |
| 4 | 0 | 16q21 | Chr16: 57,358,783 - 66,588,275 | 9229492 |
| 4 | 0 | 16q22.1 | Chr16: 66,603,874 - 68,835,537 | 2231663 |
| 4 | 0 | 16q23.3-q24.3 | Chr16: 83,719,311 - 90,092,072 | 6372761 |
| 4 | 0 | 17p11.2 | Chr17: 17,042,457 - 18,315,007 | 1272550 |
| 4 | 0 | 17p13.2-p13.3 | Chr17: 212,389 - 3,964,464 | 3752075 |
| 4 | 0 | 17q25.1 | Chr17: 74,203,582 - 74,210,655 | 7073 |
| 4 | 0 | 18q23 | Chr18: 79,679,803 - 80,247,514 | 567711 |
| 4 | 0 | 19p13.12-p13.3 | Chr19: 11,505,929 - 14,496,149 | 2990220 |
| 4 | 0 | 19q13.11 | Chr19: 32,345,594 - 34,229,288 | 1883694 |
| 4 | 0 | 20q11.21 | Chr20: 31,303,212 - 32,335,011 | 1031799 |
| 4 | 0 | 20q13.13-q13.31 | Chr20: 48,921,711 - 57,343,994 | 8422283 |
| 4 | 0 | 20q13.33 | Chr20: 61,953,469 - 64,304,820 | 2351351 |
| 4 | 0 | 22q11.22-q11.23 | Chr22: 22,513,736 - 25,231,870 | 2718134 |
| 4 | 0 | 22q12.3-q13.31 | Chr22: 36,281,280 - 47,175,699 | 10894419 |
| 4 | 0 | 22q13.32 | Chr22: 48,489,553 - 48,850,912 | 361359 |

**Supplementary table 4:** The regions with the most frequent CNV (both deletions and amplifications) occurrence, identified in MM plasma.
